# Church attendance and alloparenting: an analysis of fertility, social support and child development among English mothers

**DOI:** 10.1098/rstb.2019.0428

**Published:** 2020-06-29

**Authors:** John H. Shaver, Eleanor A. Power, Benjamin G. Purzycki, Joseph Watts, Rebecca Sear, Mary K. Shenk, Richard Sosis, Joseph A. Bulbulia

**Affiliations:** 1 Religion Programme, School of Social Sciences, University of Otago, PO Box 56, Dunedin 9054, New Zealand; 2 Centre for Research on Evolution, Belief and Behaviour, University of Otago, Dunedin 9054, New Zealand; 3 Max Planck Institute for the Science of Human History, Kahlaische Strasse 10, 07745 Jena, Germany; 4 Department of Methodology, London School of Economics and Political Science, Houghton Street, London, WC2A 2AE, UK; 5 Department of the Study of Religion, Aarhus University, Jens Chr. Skous Vej 3, Building 1451, 525, 8000 Aarhus C, Denmark; 6 Department of Population Health, London School of Hygiene and Tropical Medicine, Keppel Street, London WC1E 7HT, UK; 7 Department of Anthropology, The Pennsylvania State University, University Park, PA, 16801, USA; 8 Department of Anthropology, University of Connecticut, U-2176, Storrs, CT 06269-2176, USA; 9 Faculty of Arts, University of Auckland, Private Bag 92019, Auckland 1142, New Zealand

**Keywords:** alloparenting, ALSPAC, cooperation, fertility, ritual, social support

## Abstract

Many aspects of religious rituals suggest they provide adaptive benefits. Studies across societies consistently find that investments in ritual behaviour return high levels of cooperation. Another line of research finds that alloparental support to mothers increases maternal fertility and improves child outcomes. Although plausible, whether religious cooperation extends to alloparenting and/or affects child development remains unclear. Using 10 years of data collected from the Avon Longitudinal Study of Parents and Children (ALSPAC), we test the predictions that church attendance is positively associated with social support and fertility (*n* = 8207 to *n* = 8209), and that social support is positively associated with fertility and child development (*n* = 1766 to *n* = 6561). Results show that: (i) relative to not attending, church attendance is positively related to a woman's social network support and aid from co-religionists, (ii) aid from co-religionists is associated with increased family size, while (iii) fertility declines with extra-religious social network support. Moreover, while extra-religious social network support decreased over time, co-religionist aid remained constant. These findings suggest that religious and secular networks differ in their longevity and have divergent influences on a woman's fertility. We find some suggestive evidence that support to mothers and aid from co-religionists is positively associated with a child's cognitive ability at later stages of development. Findings provide mixed support for the premise that ritual, such as church attendance, is part of a strategy that returns high levels of support, fertility and improved child outcomes. Identifying the diversity and scope of cooperative breeding strategies across global religions presents an intriguing new horizon in the evolutionary study of religious systems.

This article is part of the theme issue ‘Ritual renaissance: new insights into the most human of behaviours'.

## 1. Introduction

At first glance, the possibility that religious rituals [1] are functional seems unlikely. Tribal dances, community sacrificial offerings and contemporary Christian church services appear to involve significant energetic, material and temporal investments that could be more directly invested in somatic maintenance, status competition and/or reproduction [2]. Yet, the ubiquity [3], persistence and historical depth of collective religious rituals are all indicative of functionality [4]. Resolving this paradox is a major challenge in the social sciences, and a substantial body of literature highlights the social functions of rituals. This literature has, however, largely not addressed how the cooperative social relationships, fostered through ritual behaviour, can directly impact biological fitness.

Scholars across several disciplines have proposed that participation in rituals mitigates selective pressures associated with group living [4–9]. By living socially, individuals attain, among other things, greater and more efficient resource production [4]. At the same time, sociality leaves individuals vulnerable to exploitation by conspecifics [10]. Under such conditions, collective resources are more likely to emerge and stabilize when individuals can reliably communicate their commitment to group goals that motivate collective action and resource production [11–16].

Specifically, participation in ritual may function as a reliable communicative signal of an individual's trustworthiness, and commitment to shared group objectives [4,6,17]. Energetic investments in ritual behaviour can, in other words, help ensure its communicative reliability and therefore protect the group, and its resources, from exploitation by outsiders. That is, in return for investments in ritual behaviour, individuals benefit materially from subsequent cooperation. Demonstrations of commitment through ritual can also serve to increase a person's status within the group [18]. Cooperative and/or status gains returned from the enactment of ritual behaviour are expected to positively impact an individual's reproductive success [19–21].

Despite empirical findings showing that ritual participation increases reputational standing [22], improves trust [23–26] and returns material benefits in the form of cooperation [27–32], studies have yet to examine how these cooperative benefits affect an individual's reproductive success [33,34]. Two critical facts make this empirical oversight all the more surprising: (i) there is a consistent positive association between an individual's frequency of ritual behaviour and her/his total offspring [35–38], and (ii) compared to our primate relatives, human mothers receive high levels of help with childrearing [39–41]. We therefore hypothesize that a key resource generated by religious groups is alloparental support to mothers [33,34]. We discuss these points in turn.

### (a) Cooperative support to mothers: from foragers to modern nation states

By virtue of earlier weaning and shorter interbirth intervals, human females achieve higher fertility compared to our great ape relatives [42–44]. The cooperative breeding hypothesis contends that high fertility among human females is accomplished, in part, through significant investments in children by individuals other than the mother (i.e. alloparents) [39,40,45–47]. Indeed, cross-cultural studies find that kin and non-kin make substantial contributions to children, and that these investments positively impact child survival and women's total fertility [41,48]. Though empirical work finds that the majority of alloparenting comes from kin, particularly maternal kin, there is considerable ecological variation associated with alloparenting and flexibility in who helps mothers [41,43,48,49].

Recent socio-economic changes (e.g. urbanization, reliance upon market economies, increased schooling and access to healthcare) have resulted in substantial reductions to human fertility [50,51], as part of the demographic transition from high fertility and high mortality to low fertility and low mortality [52]. Evolutionary anthropologists have suggested that these declines in fertility can be partially attributed to lower levels of support available to mothers in post-industrial environments [53,54]. As societies undergo economic transitions, kin networks––and the support they provide mothers––break down as individuals disperse over larger geographical ranges in pursuit of economic opportunities [46,48,55–58]. Indeed, compared to pre-industrialized populations, children in post-industrial societies receive less investment from kin, particularly older siblings, cousins, aunts and uncles [41]. However, despite an overall reduction in available support, parents in post-industrial environments invest substantially more in individual offspring than in pre-demographic transition environments [56,59].

Although there is considerable evidence for a relationship between social support to mothers and their fertility in pre-transition societies, in post-transition societies, evidence for the cooperative breeding hypothesis is more ambiguous [60,61]. Some studies find that support is positively associated with fertility [62–64], some find no relationship [65,66], while others find that social support has anti-natal effects [65,67].

Along with major economic and other social changes, post-industrial subsistence and economic patterns often also entail the emergence of secularization, and the beginnings of fertility differentials between religious and secular populations, with the former, in general, exhibiting higher fertility than the latter [33]. Although there have been several cross-cultural studies of social support and fertility across societies, few have investigated how religious and secular support networks may have different effects on a woman's fertility. Religious people tend to have higher overall levels of social support [31,68,69], the number of within-congregational social ties for mothers increases when she has a child [70], and mothers receive more social support from co-religionists than non-mothers [71]. Moreover, childless members of religious groups tend to engage in more alloparental support than their demographically similar secular counterparts [33]. It remains unknown, however, whether the higher fertility of religious individuals in post-industrial environments is a result of this greater social support.

Without higher levels of support, we would expect children born to religious parents to exhibit reduced success, owing to larger sibships and a resultant reduction or dilution of parental resources. Indeed, in post-transition societies, family size is associated with a reduction in per-child parental investment and offspring physiological and cognitive outcomes [72–75]. Both evolutionary [33] and economic theories [76–78], however, posit that religious individuals in post-industrial societies may be partially buffered from the effects of resource dilution to offspring, given higher levels of alloparental support [33]. For example, cross-culturally, sibling number is negatively related to a child's success in school, yet this trade-off is weakened, and even non-existent, among some religious groups [77,78]. It has been hypothesized that reduced trade-offs between family size and child success in school, among some religious groups, is the result of the higher relative levels of social support available to mothers in these groups [77]. To date, however, it is unknown whether higher levels of support to mothers in religious communities is sufficient to reduce the strength of trade-offs between child number and child development outcomes.

Using 10 years of data collected among mothers living in the UK, we tested the following predictions:

P1: frequency of maternal church attendance is positively associated with social network support, as well as aid from co-religionists;
P2: frequency of maternal church attendance is positively associated with a mother's fertility;
P3: social network support and aid from co-religionists are positively related to a mother's fertility, even after adjusting for church attendance; and
P4: social network support and aid from co-religionists is positively associated with child development outcomes.

## 2. Methods

### (a) Sample and participants

Here, we analyse data from the Avon Longitudinal Study of Parents and Children (ALSPAC), a panel study of focal children and their parents, living around the city of Bristol, England [79,80]. Initially, the ALSPAC sample recruited pregnant women with an expected delivery date that fell between 1 April 1991 and 31 December 1992, and who were resident in one of three health administration districts within the South-West Regional Health Authority that became the ‘Bristol & District Health Authority’. Mothers of 14 541 pregnancies were recruited prior to birth during 1990–1992, children from an additional 452 pregnancies were added to the study 7 years later, and children resulting from another 254 pregnancies were added 8–18 years later. Of these 15 257 pregnancies, 14 775 children were live-born, and 14 701 were alive at 1 year. A description of the study and the study population are available from the ALSPAC website (http://www.bristol.ac.uk/alspac/) and elsewhere [79,80].

We examine data collected from mothers and a focal child during the child's first 10 years of life. Following previous studies using the ALSPAC database [72,73], prior to analyses we removed children from multiple births, children who died or experienced sibling death, and children living with fostered or adopted children. Children who had step-siblings living in the home were included in analyses. Additional cases were removed if the mother figure was listed as absent or not the biological mother, if the mother indicated that she had no biological children living with her, if the mother ever indicated she was in a lesbian relationship, or if she was in a relationship with someone other than the biological father at the time of the focal child's birth. All other cases of biological father absence were included, though father absence was controlled for in analyses. This left data from 13 446 children and their mothers for analysis. Owing to missing data, the number of mothers and children included in models varied between 1766 to 8209, depending on the variables specified in the model and their rates of missing data.

### (b) Variables and data processing

The study website contains details of all data through a fully searchable data dictionary and variable search tool (http://www.bristol.ac.uk/alspac/researchers/our-data/). The ALSPAC codes for all variables used in the current analyses are described in the electronic supplementary material, table S1.

A difficulty in using the ALSPAC dataset is that it contains high rates of missing data. The raw dataset for our main analyses, described in table 1, contained 834 503 measured values (not including year or participant identities (IDs)), and 940 369 missing values. The greatest source of missing data in this dataset is owing to variables not being measured at the same time points. For example, the variable on mothers' social support networks is not measured in years eight or ten, whereas measurements of child's height are. Note, too, that variables tend to be measured less frequently at later time points in the study (table 1). Missing data owing to the ALSPAC study not measuring variables in a given year accounts for 631 962 (67%) of all missing data in our main dataset. This presents a challenge for multivariate models that include variables measured at different time points.

**Table 1.** Descriptive statistics of all variables used in analyses (prior to imputation; *n* = 13 446). (Percentages indicate percentage of valid cases, except for the missing cases, where the percentages are for all cases.)

year	0	1	2	3	4	5	6	7	8	9	10
*mother's fertility*											
0	5434 (45.2%)	—	0 (0%)	0 (0%)	0 (0%)	—	—	0 (0%)	—	—	0 (0%)
1	4284 (35.6%)	5434 (45.2%)	3578 (38.4%)	2015 (23.5%)	1311 (15.2%)	—	—	250 (10.3%)	—	—	212 (10.0%)
2	1702 (14.2%)	4284 (35.6%)	3859 (41.5%)	4530 (52.9%)	4906 (56.9%)	—	—	1256 (51.8%)	—	—	1069 (50.6%)
3	447 (3.7%)	1702 (14.2%)	1362 (14.6%)	1426 (16.7%)	1747 (20.3%)	—	—	615 (25.4%)	—	—	532 (25.2%)
4+	157 (1.3%)	604 (5.0%)	508 (5.5%)	587 (6.9%)	661 (7.7%)	—	—	304 (12.5%)	—	—	300 (14.2%)
missing	1422 (10.6%)	1422 (10.6%)	4139 (30.8%)	4888 (36.4%)	4821 (35.9%)	—	—	11 021 (82.0%)	—	—	11 333 (84.3%)
*mother's aid from co-religionists*											
no	9522 (90.6%)	—	—	—	—	6882 (89.9%)	6277 (88.2%)	—	—	5900 (87.6%)	—
yes	986 (9.4%)	—	—	—	—	772 (10.1%)	843 (11.8%)	—	—	834 (12.4%)	—
missing	2938 (21.9%)	—	—	—	—	5792 (43.1%)	6326 (47.0%)	—	—	6712 (49.9%)	—
*mother's partner present*											
no	687 (5.9%)	—	718 (7.7%)	221 (2.7%)	219 (2.7%)	—	—	760 (10.3%)	—	—	n.a.
yes	10 909 (94.1%)	—	8624 (92.3%)	7973 (97.3%)	7763 (97.3%)	—	—	6645 (89.7%)	—	—	6425 (100%)
missing	1850 (13.8%)	—	4104 (30.5%)	5252 (39.1%)	5464 (40.6%)	—	—	6041 (44.9%)	—	—	7021 (52.2%)
*mother's partner's children in household*											
0	11 904 (98.9%)	—	9229 (98.9%)	8459 (98.8%)	8525 (98.8%)	—	—	2118 (96.8%)	—	—	1766 (95.6%)
1	87 (0.7%)	—	56 (0.6%)	60 (0.7%)	48 (0.6%)	—	—	37 (1.7%)	—	—	44 (2.4%)
2	37 (0.3%)	—	35 (0.4%)	28 (0.3%)	40 (0.5%)	—	—	21 (1.0%)	—	—	31 (1.7%)
3+	11 (0.1%)	—	11 (0.1%)	11 (0.1%)	12 (0.1%)	—	—	13 (0.6%)	—	—	7 (0.4%)
missing	1407 (10.5%)	—	4115 (30.6%)	4888 (36.4%)	4821 (35.9%)	—	—	11 257 (83.7%)	—	—	11 598 (86.3%)
*mother's social network support*											
mean (s.d.)	23.3 (3.85)	—	23.4 (4.09)	—	—	22.2 (3.89)	22.9 (4.14)	—	—	22.7 (4.29)	—
median [min, max]	24.0 [1.00, 29.0]	—	24.0 [2.00, 29.0]	—	—	23.0 [5.00, 29.0]	23.0 [1.00, 29.0]	—	—	23.0 [1.00, 29.0]	—
missing	2457 (18.3%)	—	4284 (31.9%)	—	—	5563 (41.4%)	5894 (43.8%)	—	—	6443 (47.9%)	—
*mother's weekly hours work*											
mean (s.d.)	1.40 (5.88)	—	10.1 (13.0)	16.8 (13.1)	—	18.9 (12.5)	—	19.1 (12.5)	—	—	22.4 (13.1)
median [min, max]	0.00 [0.00, 60.0]	—	0.00 [0.00, 90.0]	16.0 [0.00, 80.0]	—	18.0 [0.00, 80.0]	—	18.0 [0.00, 72.0]	—	—	20.0 [0.00, 85.0]
missing	4474 (33.3%)	—	5077 (37.8%)	8423 (62.6%)	—	7971 (59.3%)	—	11 331 (84.3%)	—	—	12 082 (89.9%)
*mother's age*											
mean (s.d.)	28.0 (4.94)	—	—	—	—	—	—	—	—	—	—
median [min, max]	28.0 [15.0, 44.0]	—	—	—	—	—	—	—	—	—	—
missing	754 (5.6%)	—	—	—	—	—	—	—	—	—	—
*focal child sex*											
female	6539 (48.9%)	—	—	—	—	—	—	—	—	—	—
male	6835 (51.1%)	—	—	—	—	—	—	—	—	—	—
missing	72 (0.5%)	—	—	—	—	—	—	—	—	—	—
*focal child ethnicity*											
non-white	528 (4.8%)	—	—	—	—	—	—	—	—	—	—
white	10 445 (95.2%)	—	—	—	—	—	—	—	—	—	—
missing	2473 (18.4%)	—	—	—	—	—	—	—	—	—	—
*mother's weekly household income*											
<100 GBP	—	—	—	650 (8.2%)	571 (7.3%)	—	—	248 (3.6%)	—	—	—
100–199 GBP	—	—	—	1370 (17.2%)	1184 (15.2%)	—	—	729 (10.7%)	—	—	—
200–299 GBP	—	—	—	2291 (28.8%)	2054 (26.4%)	—	—	1245 (18.3%)	—	—	—
300–399 GBP	—	—	—	1705 (21.4%)	1745 (22.4%)	—	—	1554 (22.8%)	—	—	—
>400 GBP	—	—	—	1941 (24.4%)	2232 (28.7%)	—	—	3042 (44.6%)	—	—	—
missing	—	—	—	5489 (40.8%)	5660 (42.1%)	—	—	6628 (49.3%)	—	—	—
*mother's church attendance*											
never	6184 (56.4%)	—	—	—	—	4182 (53.0%)	2341 (31.2%)	—	—	3503 (49.3%)	—
yearly	3209 (29.3%)	—	—	—	—	2111 (26.8%)	3552 (47.3%)	—	—	2093 (29.5%)	—
monthly	760 (6.9%)	—	—	—	—	797 (10.1%)	806 (10.7%)	—	—	661 (9.3%)	—
weekly	816 (7.4%)	—	—	—	—	795 (10.1%)	812 (10.8%)	—	—	846 (11.9%)	—
missing	2477 (18.4%)	—	—	—	—	5561 (41.4%)	5935 (44.1%)	—	—	6343 (47.2%)	—
*mother's education*											
CSE/none	1541 (14.5%)										
vocational	1103 (10.4%)										
O level	3943 (37.2%)										
A level	2550 (24.1%)										
college degree	1455 (13.7%)										
missing	2854 (21.2%)										

Other reasons for missing data include a mother or child declining to answer a specific question, being recruited to the sample at a later time point, or being unavailable to take part in the study at a specific point in time. Such attrition can introduce biases that result in a less representative sample.

To address the methodological challenges associated with missing data and to reduce the effects of potentially biased attrition, we performed imputation for all predictor variables in the study, as well as for mother's fertility (an outcome in one of our models). Our imputation approach started by imputing missing values between known values, assuming linear change between those points. Any remaining missing values were then replaced with the nearest known recorded value. For example, if a mother was recorded as having one child in year one, and two children in year six, but no other recorded values, our method would impute one child in years two and three, and two children for any missing years thereafter. If a mother or child had no recorded values for a variable, no imputation was performed. This imputation strategy enabled us to analyse variables that are not recorded at the same time points and minimized potential biases that could arise owing to biased attrition by providing principled estimates for missing cases. To test how sensitive our findings are to this imputation strategy, we performed additional analyses without any imputation, and using an alternative imputation strategy (electronic supplementary material, methods). The results of these models are substantively identical to those of our main analyses (electronic supplementary material, table S12). The cognitive development dataset was not imputed over time because the outcome variables are tied to child performance in a single year. Instead, for these models, we assigned values to predictor variables using the last known measurement value and did not impute cognitive performance outcomes. We also did not impute measures of child height owing to the nonlinear and varying nature of child growth rates. The full code used to impute missing data is provided on the Open Science Framework (OSF) project page (https://osf.io/4mt9y/), and we provide summary tables of the raw (table 1) and imputed data (electronic supplementary material, tables S2 and S3).

#### (i) Outcome variables

We performed a series of four analyses to test each of our four predictions. Our first series of analyses examined mothers' social support using two measures––a composite measure of social network support drawn from the sum of 10 questions (see the electronic supplementary material, methods) and a binary measure of aid from co-religionists. The social network support measure was used as a proxy for general social support, and assessed a mother's potential to draw practical and emotional support, as well as frequency of contact with kin, in-laws and friends. The aid from co-religionists variable was derived from the question, ‘Do you obtain help and support from members of your religious group?’ Aid from co-religionists, therefore, represents ongoing practical aid, whereas the social network support composite variable is constructed largely from questions about the size of the potential support network, with some questions which may reflect the provision of emotional, but not practical, support. Our second series of analyses examined mother's fertility, which was operationalized as her number of offspring for each of the 10 years in the study, including those children she had at the start of the study. Our third series of analyses examined children's physiological development, operationalized as focal child height in centimetres. Our fourth series of analyses investigated child cognitive performance, operationalized with scores on compulsory national tests: upon entering school at age 5–6 (entry assessment), at age 6–7 (stage 1 assessment) and at age 8 (the WISC-III [81]). Full model specifications are outlined in the electronic supplementary materials, and the code used to perform analyses is available on the OSF project page.

#### (ii) Predictor variables

We here define ritual as ‘the performance of more or less invariant sequences of formal acts and utterances not entirely encoded by the performers' [1, p. 24]. Church services, which typically involve the recitation of formalized prayers, singing of hymns and standing and sitting at prescribed moments, are among the most frequently performed rituals across human cultures today [82].

To operationalize ritual investments, we used a four-level ordinal measure of frequency of attendance at a house of worship (none, at least once a year, monthly or weekly), which in this sample consisted mainly of Christian church attendance and is hereafter referred to as church attendance. We initially investigated the relationship between other aspects of religion, including belief and religious identification, and a mother's fertility, but found little evidence of such relationships (see the electronic supplementary material, table S11). To assess the impact of the presence of a mother's partner on her fertility, models included a dichotomous measure indicating whether or not a mother's partner resided in her household. We also included a continuous measure of mother's number of step-children in the household in relevant models. Mother's work was assessed as number of hours worked per week. All models included household income as a measure of social status. We investigated the inclusion of other status measures, but owing to concerns of multicollinearity, added only household income as it was most highly correlated with all other status measures. Mother's education (a categorical variable with five levels) was also included as a covariate as it is known to be related to her fertility. Mother's age (in years), child's ethnicity and child's sex were coded at birth and included as controls. Ethnicity was coded as 0 = for non-white ethnicity, and 1 = white ethnicity (see table 1 and the electronic supplementary material for more detail regarding ethnicity in this sample), and the focal child's sex was coded as 0 = female, 1 = male. In the models examining focal child developmental outcomes, sibling number was calculated by subtracting 1 from a mother's total fertility in each year after the child was born. Further details of all variables are provided in the electronic supplementary material, S1.2.

#### (iii) Analyses

Across all analyses we built multivariate, multilevel Bayesian models subsequently assessed with the brms package [83] in R, version 3.6.0 [84]. Participant ID and year were included as varying effects in all analyses. For our models on mother's fertility, social network support and aid from coreligionists, the number of mothers included in our analyses varied from 8207 to 8209, with a total of between 90 277 and 90 299 data points across time. For our models on cognitive performance, which are each measured at a single time point, the number of children ranged from 1766 to 5814. For our models on child height, there were 6561 children included, with a total of 29 513 height measurements recorded across time points. The size and diversity of our sample warranted the use of uninformative priors in all models. We fitted models with mother's fertility as the outcome with a Poisson distribution, models with help/support from co-religionists as the outcome with a Bernoulli distribution, and models with religious attendance, social network support, height and cognitive ability measures as outcomes with Gaussian distributions. To aid in interpretation and mixing, we mean-centred mother's age, number of step-children in her household, her weekly hours of work, her education (i.e. a one unit change in education represents a change in education level––see the electronic supplementary material) and household income (i.e. a one unit change in income represents an additional 100 GBP per week in income), and we scaled social network support (i.e. a one unit change in social support represents a standard deviation movement on the social support scale). In the height models, sibling number was centred at the mean. All data processing scripts are available on the OSF project page.

We assessed multicollinearity by building general linear models and examining the variance inflation factors (VIFs) for each predictor in the models. All VIFs were less than 1.4, indicating that multicollinearity was not an issue [85]. For all models, we ran five chains at 2000 iterations per chain, with the first 500 iterations set as a warmup period. All Ř values were between 1.00 and 1.02, indicating chain convergence in all models. Model diagnostics are available on the project's OSF page.

We performed follow-up analyses investigating the interaction between mothers' frequency of church attendance and each of the social support measures for our models predicting mothers’ fertility, child cognitive performance and child height. The results of these models are provided in the electronic supplementary material tables S5–S6 and S8–S10.

## 3. Results

Of the 13 446 mothers who comprised our dataset, at the birth of the focal child, 9396 (70%) reported a religious affiliation, and 1680 (12%) reported no affiliation (with missing data from the remaining 2370 mothers). Most religious women belonged to the Church of England (7141; 53%), with the second and third largest groups reporting Catholic (925; 7%) or other Christian (772; 6%) affiliations. We focus here on the relationship between church attendance and social network support, aid from co-religionists, and a child's height and cognitive ability. In each of the following sections, we discuss how the results of our models relate to each of our hypotheses.

### (a) Is frequency of maternal church attendance positively associated with a mother's social network support, and/or aid from co-religionists?

**Figure 1.**
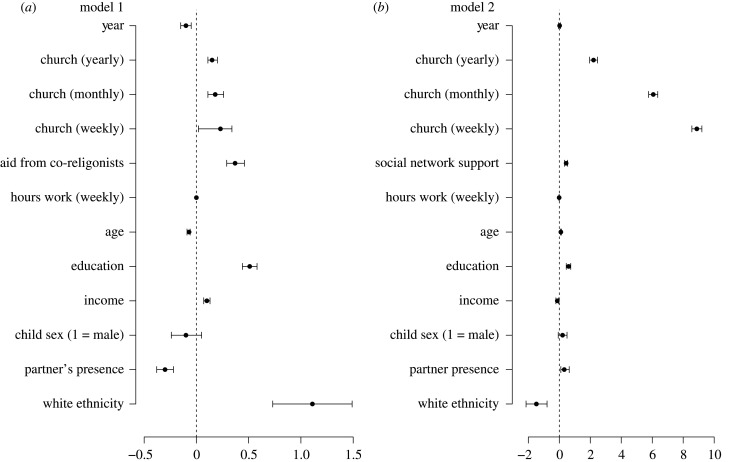
Results of models predicting mother's social network support (*a*), and aid from co-religionists (*b*). Coefficients include 95% confidence intervals. Note: social network support is scaled in model 2.

Time since the focal child's birth year was associated with a decrease in a mother's social network support (−0.10, 95% confidence intervals (CI) = [−0.15, −0.05]), indicating that mother's social network support decreased as the focal child aged (electronic supplementary material, table S4, model 1; figure 1*a*). Yearly (0.15, 95% CI = [0.11, 0.20]), monthly (0.18, 95% CI = [0.11, 0.26]) and weekly (0.23, 95% CI = [0.02, 0.34]) church attendance were all related to greater support relative to mothers who never attend church, though there were no meaningful differences between these different levels of church attendance and social network support. Mothers' education (0.51, 95% CI = [0.44, 0.58]), household income (0.10, 95% CI = [0.07, 0.13]) and ethnicity (white; 1.11, 95% CI = [0.73, 1.49]) were also associated with increased social network support. Mother's age (−0.07, 95% CI = [−0.09, −0.06]) and partner presence (−0.30, 95% CI = [−0.38, −0.22]) were negatively associated with social network support. There was no evidence of an association between the sex of the focal child (−0.10, 95% CI = [−0.24, 0.05]), or working hours (0.00, 95% CI = [0.00, 0.00]) on mothers' social network support.

In contrast with social network support, time since the focal child's birth was not associated with a change in the likelihood of aid from co-religionists (0.02, 95% CI = [−0.05, 0.08]) (electronic supplementary material, table S4, model 2; figure 1*b*), indicating that as focal children aged, help to religious mothers' remained consistent. Holding all else constant, church attendance at the yearly (2.21, 95% CI = [1.96, 2.47]), monthly (6.07, 95% CI = [5.77, 6.36]) and weekly (8.88, 95% CI = [8.56, 9.21]) levels were all associated with an increase in the probability of mothers receiving aid from other members of their religious group, when compared to mothers who never attended church. A mother's age (0.10, 95% CI = [0.07, 0.14]) was positively associated with aid from co-religionists, while being an ethnic minority (−1.48, 95% CI = [−2.15, −0.79]), and household income was negatively associated with aid from co-religionists (−0.13, 95% CI = [−0.22, −0.04]). Partner presence was associated (0.32, 95% CI = 0.10, 0.64]) with an increased likelihood of co-religionist aid.

Together, these results suggest that the more frequently mothers attend church, the greater their social network support and the more likely they are to receive aid from co-religionists.

### (b) Is the frequency of maternal church attendance positively associated with fertility?

**Figure 2.**
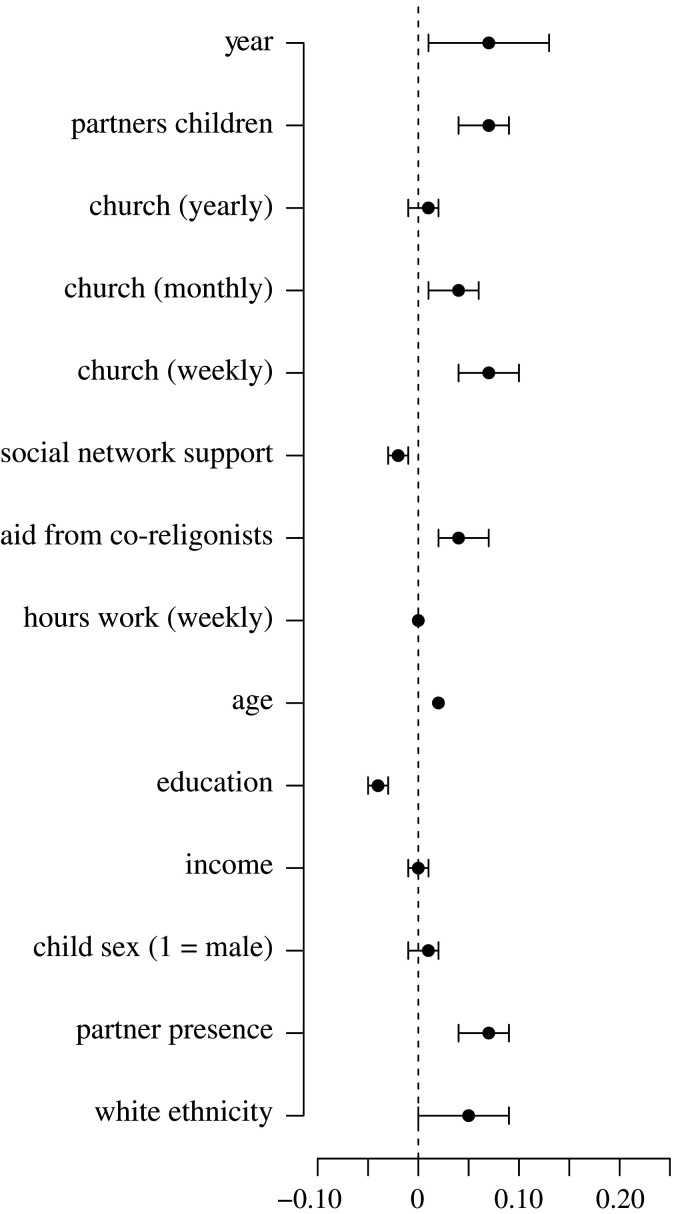
Results of models predicting a mother's fertility. Coefficients include 95% confidence intervals.

Mother's monthly (0.04, 95% CI = [0.01, 0.06]) and weekly (0.07, 95% CI = [0.04, 0.10]) church attendance were both associated with an increase in her fertility, though there was no reliable association between attending church yearly and a woman's fertility (0.00, 95% CI = [−0.01, 0.02]) (electronic supplementary material, table S5, model 1; figure 2). Mother's education (−0.04, 95% CI = [−0.05, −0.03]) was negatively associated with her fertility. The presence of a mother's partner (0.07, 95% CI = [0.04, 0.09]), presence of partner's children (0.18, 95% CI = [0.15, 0.20] and mother's age (0.02, 95% CI = [0.02, 0.02]) were all positively associated with her fertility. With an average age of 28 and a s.d. of less than 5, and with sample participants moving through peak (Western) fertility over the decade under study, we expect a greater effect from time (0.07, 95% CI = [0.01, 0.13]), than mother's age at the focal child's birth, which is what we observe here. A mother's household income (0.00, 95% CI = [−0.01, 0.01]), her working hours (0.00, 95% CI = [0.00, 0.00]) and the focal child's sex (0.01, 95% CI = [−0.01, 0.02]) were not reliably associated with changes in a woman's fertility. Women of white ethnicity tended to have more children (0.05, 95% CI = [0.00, 0.09]).

These results provide support for the hypothesis that religious attendance is positively associated with a mother's fertility.

### (c) Is a mother's social network support, and/or aid from co-religionists positively related to her fertility?

Aid from co-religionists was positively associated with a mother's fertility (0.04, 95% CI = [0.01, 0.06]), while the measure of mothers' social network support was negatively associated with her fertility (−0.02, 95% CI = [−0.03, −0.01]) (electronic supplementary material, table S5, model 1; figure 2).

**Figure 3.**
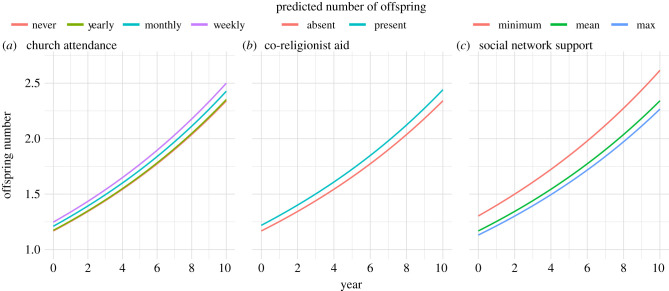
Key predictors of mothers fertility over 10 years of the study: (*a*) illustrates predicted number of children among mothers by mother's church attendance; (*b*) illustrates the predicted number of children depending on whether mothers receive aid from co-religionists; and (*c*) illustrates predicted number of children by social network support; minimum shows the predicted number of children for the lowest observed level of social network support found in our dataset; mean illustrates the predicted number of children for the mean level of social network support observed in our dataset, and maximum illustrates the predicted number of children for the highest level of social network support observed in our dataset. All three of these figures are based on the coefficients estimated electronic supplementary material, table S5.

Results indicate that mothers who receive more help from co-religionists tend to have more children than mothers who receive less help from co-religionists. How large is the effect of alloparental support from co-religionists on fertility? Figure 3*b* plots the predicted effects of aid from co-religionists over time. In order to understand these effects in practical terms, we can compare the expected effect of aid from co-religionists when all other model terms are set to their mean. Over 10 years, women can expect to have 2.36 children. Women who attend church weekly are expected to have 2.53 children, and among them, those who receive aid from co-religionists are expected to have 2.64 children.

### (d) Is a mother's social network support and/or aid from co-religionists positively associated with child physiological and cognitive development?

Neither a mother's social support (0.01, 95% CI = [−0.08, 0.05]) nor aid from co-religionists (−0.14, 95% CI = [−0.31, 0.03]) was related to a child's height (electronic supplementary material, table S6; model 1). Replicating previous findings [72], models show a negative association between a child's number of siblings and the child's height (−0.24, 95% CI = [−0.32, −0.16]). There was no association between the number of step-siblings in the household and a child's height (0.10, 95% CI = [−0.11, 0.31]).

There were small but discernable associations between a child's height and a mother's yearly (0.11, 95% CI = [0.01, 0.21]) and monthly (0.16, 95% CI = [0.00, 0.32]), but not weekly church attendance (0.19, 95% CI = [−0.03, 0.42]). There was a small positive relationship between a mother's hours of work and a child's height (0.01, 95% CI = [0.01, 0.02]). Children of white ethnicity (−0.92, 95% CI = [−1.59, −0.23]) tended to be shorter. There was also a negative association between a mother's household income and child height over time (−0.08, 95% CI = [−0.15, −0.01]). A mother's education was not related to her child's height (−0.07, 95% CI = [−0.19, 0.05]), nor was there an association between a mother's partner's presence and a child's height (0.18, 95% CI = [−0.02, 0.38]). Mother's height was positively associated with child height (0.32, 95% CI = [0.30, 0.34]), and boys grew taller than girls (0.71, 95% CI = [0.47, 0.96]).

**Figure 4.**
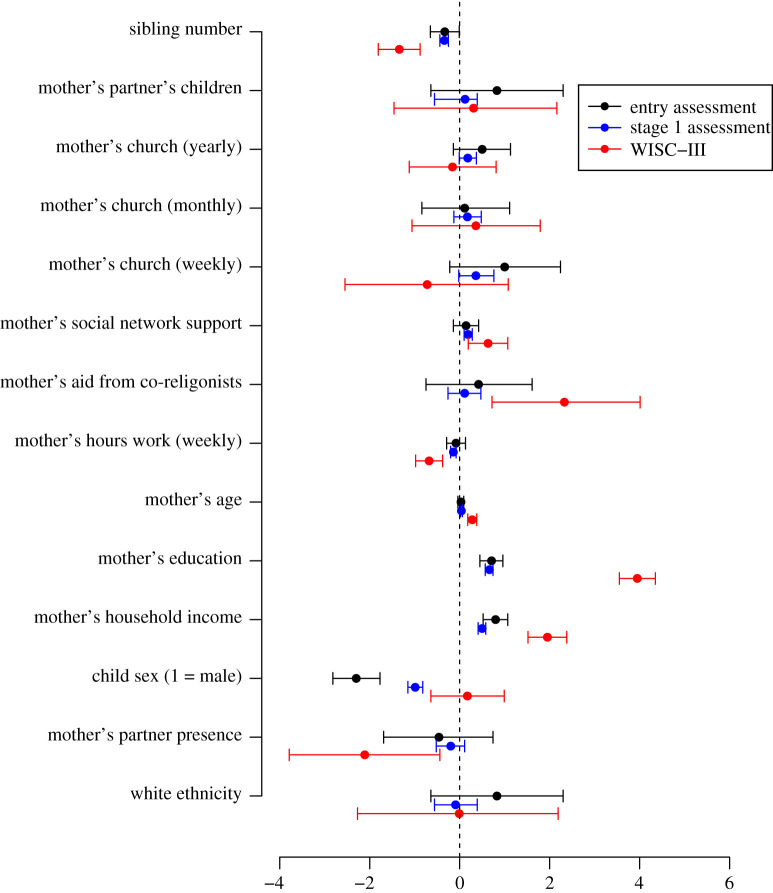
Results of models predicting a child's performance on three standardized measures of cognitive ability. Coefficients include 95% confidence intervals. (Online version in colour.)

In terms of cognitive development measures, a mother's social support was not related to a child's entry level assessment (0.14, 95% CI = [−0.14, 0.42]), but was positively related to scores on both the stage 1 assessment (0.19, 95% CI = [0.10, 0.28]) and the WISC-III (0.63, 95% CI = [0.19, 1.07]) (electronic supplementary material, table S7; figure 4). There was no relationship between aid from co-religionists to mothers and a child's entry level assessment (0.42, 95% CI = [−0.75, 1.61]), nor the stage 1 assessment (0.11, 95% CI = [−0.26, 0.47), but there was a positive association between aid from co-religionists and the WISC-III (2.33, 95% CI = [0.72, 4.01]). There was a negative effect of sibling number on entry assessment scores (−0.33, 95% CI = [−0.65, −0.01]), on the stage 1 assessment (−0.34, 95% CI = [−0.44, −0.25]) and the WISC-III (−1.34, 95% CI = [−1.81, −0.88]). There was no evidence of a relationship between step-siblings in the household and a child's score on the entry level assessment (0.83, 95% CI = [−0.64, 2.30]), stage 1 assessment (0.12, 95% CI = [−0.56, 0.39]), nor the WISC-III (0.31, 95% CI = [−1.46. 2.16]).

The number of hours a mother spent working was not reliably related to a child's performance on the entry level assessment (−0.08, 95% CI = [−0.29, 0.13]), but was negatively related to a child's stage 1 assessment (−0.14, 95% CI = [−0.20, −0.08]) and WISC-III (−0.68, 95% CI = [−0.98, −0.38]) scores. A mother's education was positively related to a child's entry level assessment (0.71, 95% CI = [0.45, 0.96]), stage 1 assessment (0.66, 95% CI = [0.57, 0.74]) and WISC-III (3.95, 95% CI = [3.55, 4.35]). Household income was also positively related to child's entry level assessment (0.80, 95% CI = [0.52, 1.07]), stage 1 assessment (0.50, 95% CI = [0.41, 0.58]) and WISC-III (1.95, 95% CI = [1.52, 2.39]). Boys scored lower on entry level assessments (−2.30, 95% CI = [−2.82, −1.77]) and stage 1 assessments (−0.99, 95% CI = [−1.15, −0.82]), but there were no differences between the sexes on the WISC-III (0.17, 95% CI = [−0.64, 0.99]). The presence of mother's partner in the home did not influence a child's entry level (−0.46, 95% CI = [−1.69, 0.74]) or stage 1 assessment (−0.20, 95% CI = [−0.52, 0.11]), though there is evidence of a negative relationship between mother's partner's presence and a child's WISC-III score (−2.11, 95% CI = [−3.79, −0.44]). Children of older mothers scored higher on the WISC-III (0.28, 95% CI = [0.18, 0.38]) and the stage 1 assessment (0.04, 95% CI = [0.02, 0.06]), but there is no evidence of a relationship between a mother's age and a child's performance on the entry level assessment (0.03, 95% CI = [−0.04, 0.09]). There was no relationship between a child's ethnicity and any of the three test scores.

Together, these findings provide only limited support for our hypotheses. Most models showed no relationships between ritual attendance, aid from co-religionists or social support and child outcomes. However, when associations were found, all were in the predicted direction, which provides some suggestive evidence to support our hypotheses: aid from co-religionists and mother's social network support were positively associated with small differences in performance on measures of cognitive development at later stages of child maturity. The results showing positive associations between church attendance and child height are difficult to interpret, given that aid from co-religionists was not associated with height.

## 4. Discussion

Our results suggest that church attendance is positively associated with social support and aid from co-religionists, and that aid from co-religionists is associated with increased fertility and may also be associated with improvements in some child outcomes. We caution that the findings reported here do not definitively establish causality. Rather, the observations are consistent with the hypothesis that engaging in religious rituals helps build social support networks with co-religionists, and that support from co-religionists increases fertility and may improve some aspects of child development.

Although fertility levels in post-industrial populations are known to vary with religious group membership and ritual participation, these relationships have rarely been subjected to sustained evolutionary theorizing and empirical investigation [86–89]. We predicted that higher relative fertility among religious individuals in post-industrial environments is, in part, enabled by greater alloparental support available to mothers in these communities. Specifically, we predicted that mothers who attend church more frequently benefit from larger and higher quality social networks, and aid from co-religionists, and that increased support is associated with larger family sizes over time. Indeed, we found an overall positive relationship between church attendance and both social support and aid from co-religionists, and a positive relationship between aid from co-religionists and a woman's fertility. Though church attendance was associated with higher levels of social network support, we did not find reliable differences in social support among mothers who attended at different frequencies. Additionally, we found an overall negative relationship between a mother's social network support and her fertility. These findings suggest that religious and non-religious social networks may have opposing influences on fertility in line with some previous findings [53,54]. However, how religious and secular networks might differ, and why they have different relationships with fertility beyond alloparental support, remain unclear—and our ability to further disentangle these relationships with the current data is limited.

Evolutionary theorists have predicted that human reproductive decision-making evolved to facultatively respond to local levels of available alloparental support [74]. However, the specific features of social support that influence fertility decisions remain unclear, and indeed, different kinds of social support have been found to differentially affect fertility. For example, among a UK sample, emotional support was found to have a positive association with transitions to a second child, while practical support was negatively related to second births [60]. Other studies have found associations between transitions to second births and frequency of contact with family members [90] and in-laws [91]. Recent work has found that in addition to kin support, the likelihood of receiving alloparental support is influenced by having given support [90] (i.e. alloparenting is often reciprocal). In the current study, social support is measured through questions that assessed a mother's potential to draw practical and emotional support, as well as frequency of contact with kin, in-laws and friends. We found an overall negative association between this measure of social support and fertility. Scholars have suggested that a negative relationship between support and total fertility in post-industrial environments may be the result of women with young children using social support to return to work, rather than to have more children [92]. However, we found that a negative relationship between social support and fertility holds after adjusting for mother's working hours. It is possible that over time mothers with more children withdraw socially in order to focus on childrearing; indeed, we found that mothers in the ALSPAC sample had lower levels of social support as the focal child aged. That is, rather than high levels of social support affecting declines in fertility, the negative relationship between social support and fertility we find here may be the result of mothers' greater focus on childrearing, at the expense of sociality, as her children age. By contrast, aid from co-religionists remained consistent over time, which suggests that relative to secular mothers, religious mothers retain higher levels of sociality, and support from their social groups, as their children age.

In post-industrial environments, increased family size is associated with decreased child development outcomes [72–74,93]. The ALSPAC data analysed here have previously been used to demonstrate a trade-off between number of offspring and offspring developmental outcomes [72,94], and to show that parental investment in a child is inversely related to a child's number of siblings [73]. If the trade-off between offspring outcomes and offspring quantity is owing to a dilution of parental resources, then parents who can draw resources from larger and stronger support networks can be expected to be partially buffered from these trade-offs. Our models reveal a negative relationship between sibling number and a child's height and performance on cognitive assessments, and that social support to mothers and aid from co-religionists is positively associated with some scores on cognitive tests at later stages of development. These findings are by no means conclusive, given that not all of our models showed the predicted positive relationship between support and child outcomes, but do provide suggestive evidence that mother's support networks may buffer the detrimental effects of sibling number on child development.

### (a) Limitations of the present study

We acknowledge that there are a number of limitations in this study. First, we emphasize that we do not treat the cognitive ability measures we analysed here as truly objective measures of a child's success. The measures of cognitive ability we used are standardized tests that are known to contain ethnic and socio-economic biases that favour white and wealthy children [95,96], and indeed our models reveal the effects of a child's household income and mother's education on a child's scores (though we find no evidence of an ethnic bias). Despite such biases, these scores may predict a child's success in the particular environment they are administered, and where similar social biases persist throughout a child's life. Research suggests that scores of cognitive ability are positively associated with later educational success [97,98] and adult socio-economic status, even after adjusting for ethnicity and the socio-economic status of the household in which a person was raised [99]. In other words, our findings suggest that the larger relative social resources available to religious mothers in England may positively impact a child's socio-economic success into adulthood. Indeed, social success is probably more fitness relevant than small differences in individual height.

Second, it is likely that other features of religions beyond social support also affect offspring outcomes among religious mothers; specifically, religious mothers may be able to provide more parental investment than secular mothers. In Westernized settings, the parents of religious children are more likely to stay married [100] and be more cooperative in childcare [101]. Cross-culturally, religious women are less likely to work than secular women [102]. These factors suggest that religious women may have more time and resources to invest in children than secular mothers; however, whether they do, and how this impacts child outcomes, remains an open question. The ALSPAC data used in this study do not allow for an examination of the influence of traditional gender norms in fertility decisions. However, we find that after adjusting for number of mother's hours of work, religious mothers still have more children, which suggests that more than traditional gender norms regarding mothers' work/domestic life are driving the findings reported here.

Third, in addition to social support and parental investment, religious and secular social networks differ in many ways that may proximately influence fertility differentials. Many religions are pro-natal, and internalization of these values could increase fertility [103]. Individuals in secular social networks, by contrast, often endorse anti-natal views that could decrease fertility [54,104]. Moreover, differences in secular and religious norms related to abortion [105], ideal family size [106] and contraception [107] may influence differences in fertility between the religious and non-religious.

Fourth, it is possible that the positive relationship between aid from co-religionists and family size we find here is driven by increased exposure to pro-natal norms rather than by alloparental support to mothers. Because of the lack of assessment of norms related to family size in the ALSPAC study, however, we cannot directly examine the relative effects of pro/anti-natal norms on mothers’ fertility in this sample. Yet if our findings were driven by norms, and exposure to pro-natal norms occurs primarily in the context of ritual (through religious teachings, for example), then we would not expect to find the additional effects of aid from co-religionists on fertility reported above. Rather than representing an alternative set of hypotheses, it could be that both normative differences between religious and secular mothers as well as differences in practical support could be influencing differences in fertility between these groups. If the higher relative fertility of religious individuals is driven primarily by norms, however, they are unlikely to provide any buffer to offspring development without concomitant norms for cooperative support to mothers. Indeed, the effects of religious/secular norms on fertility, and how these may affect family size relative to, or in conjunction with, cooperative support remains a critical area for future research.

Fifth, a potential issue with analyses that test whether support (from kin, non-kin or co-religionists) is associated with improved child outcomes is that they assume that all mothers are the same except that some have access to additional support while others do not. Instead, it may be that mothers who receive support from co-religionists are compensating for lack of support, or other disadvantages, in other areas of their lives. In other words, a lack of evidence that support improves child outcomes does not necessarily mean that support does not improve child outcomes––children may have been worse off in the absence of that support, if their mothers belong to a particularly disadvantaged group.

Sixth, there is panel attrition in the ALSPAC data, which could, in theory, bias results. Specifically, for attrition to bias our results it would need to be uneven with respect to mechanisms we posit here: i.e. religious mothers who receive social support but have fewer children would need to drop out of the study at a higher rate than non-religious mothers and religious mothers who do not receive social support and yet have more children. Previous analyses have concluded that women with complications at the focal pregnancy, those of low socio-economic status (particularly those with inadequate housing), and those with low levels of social support were more likely to drop out of the study [80]. Thus, attrition owing to these inequalities resulted in a less representative sample, and with respect to social support, our findings may underestimate the effects of social support on fertility, as most women in the dataset we analysed here had relatively high levels of social support. Selective attrition also probably results in an underestimation of later educational outcomes for children [108], which indicates that the negative effect of sibling number on cognitive performance may be greater than our models are able to recover.

Finally, the social support measures used in this study do not disentangle support from kin from support from non-kin. This ambiguity limits our ability to test key evolutionary hypotheses regarding kin versus non-kin effects on fertility. Nonetheless, our findings are consistent with the hypothesis that women who attend church receive more alloparental support than secular mothers, and this support is associated with higher fertility and, on some measures, improved child outcomes.

## 5. Conclusion

In conclusion, we tested the hypothesis that one of the functions of church attendance in post-demographic transition societies is to support a high fertility/high child outcome strategy. We found mixed support for our predictions. Our analyses of the ALSPAC data suggest that: (i) church attendance is associated with more social network support and more practical help from co-religionists, (ii) help from co-religionists is associated with higher fertility, and (iii) social network support is negatively associated with total fertility. The support mothers draw upon is positively associated with some measures of cognitive ability at later child ages. To extend these findings, we encourage researchers to investigate multiple measures of support available to mothers, to study how different forms of support influence reproductive decision-making and child development, and to investigate the relative role of norms for family size and alloparental cooperation among and between religious and secular groups. Understanding the relationships between religion, support, fertility, and child development is key to understanding the function of ritual in post-industrial environments and the prevalence of religion in the future.

## Supplementary Material

Description of measures and results

## Data accessibility

Data used for this submission will be made available on request to the executive (alspacexec@bristol.ac.uk). The ALSPAC data management plan describes in detail the policy regarding data sharing, which is through a system of managed open access. R scripts for all analyses are publicly available on the Open Science Framework (https://osf.io/4mt9y/).
