# Supplementary material for: Church attendance and alloparenting: an analysis of fertility, social support and child development among English mothers
Source: Philos Trans R Soc Lond B Biol Sci. 2020 Jun 29;375(1805):20190428. doi: 10.1098/rstb.2019.0428 (PMC7423262; doi:10.1098/rstb.2019.0428)
Supplement: Description of measures and results [file rstb20190428supp1.docx]

**Supplementary Materials**

**Shaver et al., “Church attendance and alloparenting: An analysis of fertility, social support, and child development among English mothers”**

*Contents Pages*

1. Supplementary Methods …………………………………………………………………………. 2 - 5
2. Supplementary Results ……………………………………………………………………………. 6 - 23
3. Supplementary References ……………………………………………………………………… 24

**1. Supplementary Methods**

- 1. *Testing alternative imputation strategies*

We performed two follow up analyses to test how the imputation strategy used in this paper effects inference. Statistical models implemented the same software package as our main analyses (brms in R), used the same settings, and tested our predictions about the factors associated with differences in mother’s fertility. The full code for these models, and their results, are available on the Open Science Framework (OSF) project page: <https://osf.io/4mt9y/>

- - 1. *Follow up model 1: no imputation*

In the first follow-up model, we do not impute any missing data (Supplementary Table 12). This provides a test whether the the findings of our models are an artefact of our imputation strategy. Due to differences in the time points at which variables are measured, and the relatively high rates of missing data in some control variables, we only included the variables central to our predictions. These variables are mother’s church attendance, aid from co-religionists, and social network support. We also note that it was only possible to test the impact of these predictor variables in Year 0 of the study due to the mismatch between the time at which fertility and our key predictor variables are measured. This yields a total number of 10,014 participants in our unimputed dataset. Because this model did not include repeated measures of subjects, we did not include year or participant ID as random effects in this model.

Consistent with the results reported in our main analyses, this model using non-imputed data indicate that mothers that received help from co-religionists had higher fertility, and social support was negatively associated with mothers fertility in general (Supplementary Table 12). Also consistent with the results of our main analyses, we find that monthly and weekly, but not yearly, religious attendance are associated with greater fertility. The estimated effects of this model are all greater than those found in the results reported in the main article, likely due to the lack of additional control variables. While this model of unimputed data cannot incorporate the control variables, and cannot include the effects of time, the findings are consistent with the main models reported in our text.

- - 1. *Follow up model 2: Alternative imputation*

In the second follow-up model, we performed an alternative imputation strategy using the R package Amelia II (Honaker, King, & Blackwell, 2011). This provides a test of whether the inferences we report could be biased by the specific imputation strategy that we implemented. The Amelia software package uses a bootstrap Expectation-Maximization (EM) algorithm to provide computationally efficient imputation of time series data. Unlike the data we report on in the main analyses, we also imputed the outcome variable (mother’s fertility) for this dataset. The only bounds we placed on the imputed values were that mother’s total fertility must be between one and the maximum number of observed children in the dataset (22). This imputed dataset included 13,433 mothers, and 147,364 imputed and observed data points of the outcome variable (mother’s fertility). Due to the computationally intensive nature of our Bayesian models, we only imputed a single dataset in Amelia, using the same seed values as those used throughout this paper.

When modelling the data imputed in Amelia, we used the same mixed effects model structure as is reported in Supplementary Table 12, as well as the same settings. This means that any differences between the results of these models are due to the differences between imputation strategies. The results of these two imputation approaches are highly similar (Supplementary Table 12). The only notable difference in the results of these models is that the estimated effects of mothers religious attendance are all slightly higher in the models imputed in Amelia than in our main model, and that the 95% CI for yearly attendance does not cross 0 (Supplementary Table 12). Unlike the results reported in the text, this finding suggests that yearly church attendance is associated with greater fertility (in addition to the effect of weekly and monthly church attendance, consistent with the results reported in our main text). These results indicate that our findings hold across substantially different imputation methods.

In summary, the results of analyses without imputation and using an alternative imputation strategy are both highly consistent with the finds we report in the main article.

*1.2. Measures used in analyses*

| Below we list all variables used in this study, along with a description of its origin and processing. | |
| --- | --- |
| **Mother’s age** | Mother's age was collected at the time of the focal child's birth. |
| **Mother’s Social Network Support** | Mother's social network support was assessed using a composite measure from 10 variables collected at gestation, 1 year 9 months, 5 years 1 month, 6 years 1 month, and 9 years 1 month.  The 10 questions used to assess social network size/quality include: 1) number of mothers/partners relatives seen at least twice a year, 2) Number of friends mother has, 3) Mother belongs to a close circle of friends, 4) Number of people including partner mother can talk to about problems, 5) Number of people who talk to mother about their problems, 6) Number of people mother can discuss important decisions with, 7) Number of people mother can borrow 100 GBP, 8) number of people who would help mother in times of trouble, 9) Number of times mother got together with friends in last month, 10) Number of times mother got together with relatives in last month. |
| **Mother’s aid from co-religionists** | A dichotomous measure assessing whether or not a mother receives help and support from a co-religionist collected at gestation, 5 years 1 month, 6 years 1 month, and 9 years 1 month. This question specifically asked mothers, “Do you obtain help and support from members of your religious group?” |
| **Mother’s Partner present** | A dichotomous measure indicating whether or not a mother's partner resided in her household was recorded at 1 year 9 months, 2 years 9 months, 3 years 11 months, 7 years 1 month, and 10 years. At 10 years the partner presence variable only indicates those mother’s who have a partner, and not those who do not. |
| **Household Income** | We used household income as a measure of social status. Household income was coded as 1 = <100 GBP; 2 = 100-199 GBP; 3 = 200-299 GBP; 4 = 300-399 GBP; 5 = >400 GBP per week, and was assessed at years 2 years 9 months, 3 years 11 months, and 7 years 1 month. |
| **Mothers fertility** | Total number of children was assessed at gestation, 1 year 9 months, 2 years 9 months, 3 years 11 months, 7 years 1 month, and 10 years. |
| **Step-children** | Total number of step-children in the household (children of mother’s partner) was assessed at gestation, 1 year 9 months, 2 years 9 months, 3 years 11 months, 7 years 1 month, and 10 years. |
| **Sibling Number** | Total sibling number was created by subtracting 1 from mother’s overall fertility, after the focal child was born. |
| **Church attendance** | Mother's church attendance was collected at gestation, 5 years 1 month, 6 years 1 month, and 9 years 1 month, and was derived from the question, “*Do you go to a place of worship?”* Mother's church attendance was coded as 0 if they never attend, 1 if they attend yearly, 2 if they attend monthly and 3 if they attend weekly. When church attendance was assessed at 6 years 1 month, an additional option of "only on special occasions" was added to the question. "Only on special occasions" was coded as 1, to keep the measurement intervals consistent with the other measurement years. Although church attendance among the majority of the sample entails attending church, some participants are the members of other religions (e.g., Buddhist, Jewish, Rastafarian). |
| **Mother's Education** | Mothers was coded as: 1 = CSE/none; 2 = Vocational; 3 = O level; 4 = A level; 5 = College degree, and was assessed at gestation the focal child's birth. |
| **Mother’s hours of weekly work** | Mother's work per week was assessed in hours at gestation, 2 years, 3 years, 5 years, 7 years and 10 years. |
| **Focal child ethnicity** | Ethnicity of the mother and father was coded at the focal child’s birth. The overwhelming majority of mothers and fathers in the original sample identified as white (97.7% of mothers and 97.4% of fathers). The largest ethnic minority groups among mothers were Black Caribbean (0.07%), Indian (0.05%) and “other” (0.05%). Children in the study were coded as non-white if either parent reported a non-white ethnicity, and this code (white/non-white) was used in analyses reported in the text. |
| **Focal child sex** | The focal child's sex was recorded at birth. A coding of 1 indicates that the child is male, and 0 indicates the child is female. |
| **Focal child’s height** | The focal child's height was measured (in centimeters) at 4 months, 8 months, 12 months, 18 months, 25 months, 31 months, 37, months, 43 months, 49 months, 61 months, 7 years 1 month, 8 years 1 month, 9 years 1 month, and 10 years. |
| **Mother's height** | Mother's height was assessed at the focal child's birth and measured in centimeters. |
| **Cognitive Measure 1: Entry Assessment** | Entry Assessment is a compulsory national test administered when a child was 4 or 5, and prior to the child starting school. This assessment attempted to capture ability in reading, writing, mathematics and language skills. The variable we analysed summed entry assessment scores across all four areas, and scaled them by a factor of 4 and rounded to the nearest integer (see ALSPAC documentation for further detail). |
| **Cognitive Measure 2: Stage 1** | Stage 1 Assessment is a compulsory national test administered when a child was 6 or 7, at the end of the second year of schooling. This assessment attempted to capture ability in reading, writing, mathematics. Scores across all three Stage 1 Assessment areas were summed, and scaled by a factor 3 and rounded to the nearest integer (see ALSPAC documentation for further detail). |
| **Cognitive Measure 3: The WISC-III^UK^** | The WISC-III^UK^ is comprised of a “verbal” section and a “performance” section. The verbal section includes questions that assess a child’s vocabulary, but also questions that require verbal arithmetic to solve. The performance section involved assessing pattern recognition, puzzle solving and spatial memory, among other task. In the analyses presented in the paper, a variable was used which summed a child’s score across all components of the ISC-III. |
| **Mother’s Religious Affiliation** | Mother’s religious affiliation (yes or no) was collected at gestation, 5 years 1 month, 6 years 1 month, and 9 years 1 month. This variable was only included for the purpose of preliminary analyses, presented in Table S11. |
| **Mother’s Religious Belief** | Mother’s religious belief (no, unsure, yes) was collected at gestation, 5 years 1 month, 6 years 1 month, and 9 years 1 month. This variable was only included for the purpose of preliminary analyses, presented in Table S11. |

**2. Supplementary Results**

| **Table S1: ALSPAC variables used in analyses** | | | | | | | | | | | |
| --- | --- | --- | --- | --- | --- | --- | --- | --- | --- | --- | --- |
|  | **Year 0** | **Year 1** | **Year 2** | **Year 3** | **Year 4** | **Year 5** | **Year 6** | **Year 7** | **Year 8** | **Year 9** | **Year 10** |
| **Mother’s offspring** | a530, a531 | - | g530, g531 | h410, h411 | j382, j383 | - | - | m3070, m3071 | - | - | q3070, q3071 |
| **Mother’s aid from co-religionists** | d818 | - | - | - | - | k6249 | l7051 | - | - | p4051 | - |
| **Partner present** | a522 | - | g506 | h481 | j601 | - | - | m3202 | - | - | q3011 |
| **Partner’s children in household** | a532 | - | g532 | h412 | j384 | - | - | m3072 | - | - | q3072 |
| **Social network support** | d780 | - | g240 | - | - | k8000-k8009 | l7000-l7009 | - | - | p4000-p4009 | - |
| **Mother's weekly hours work** | e520, e523 | - | g674, g768 | h674, h675 | - | k6002, k6006 | - | m5007, m5012 | - | - | q5007, q5012 |
| **Mother's age** | mz028b | - | - | - | - | - | - | - | - | - | - |
| **Focal child sex** | kz021 |  | - | - | - | - | - | - | - | - | - |
| **Ethnicity** | c804 | - | - | - | - | - | - | - | - | - | - |
| **Weekly household income** | - | - | - | h470 | j410 | - | - | m5180 | - | - | - |
| **Mother’s church attendance** | d816 | - | - | - | - | k6247 | l7049 | - | - | p4049 | - |
| **Mother's education** | c645 | - | - | - | - | - | - | - | - | - | - |

Height was measured at 4 months (cf050), 8 months (cf051), 1 year (cf052), 1 year and 6 months (cf053), 2 years and 1 month (cf054), 2 years and 7 months (cf055), 3 years and 1 month (cf056), 3 years and 7 months (cf057), 4 years and 1 month (cf058), 5 years and 1 month (cf059), 7 years (f7ms010), 8 years (f8lf020), 9 years (f9ms010), 10 years (fdms010). Child cognitive development was measured at the Entry Level Assessment (sat092b), The Stage 1 Assessment (sat190b), and the WISQ-III (f8ws112).

| **Table S2: Descriptive statistics of all variables used in main analyses after imputation.** The total number of mothers included in this dataset is 13,446 and there are a total of 2,560,794 non-missing data points for the variables in the table below. The values reported in this table are before exclusions were made due to missing values in the variables needed to run specific models and the number of mothers included in each model is included in the descriptions of Tables S4, S5 & S7-S11.Percentages indicate percentage of valid cases, except for the missing cases, where the percentages are for all cases. | | | | | | | | | | | |
| --- | --- | --- | --- | --- | --- | --- | --- | --- | --- | --- | --- |
| Year | **Year 0** | **Year 1** | **Year 2** | **Year 3** | **Year 4** | **Year 5** | **Year 6** | **Year 7** | **Year 8** | **Year 9** | **Year 10** |
| **Mother’s fertility** | |  |  |  |  |  |  |  |  |  |  |
| 0 | 5434 (44.0%) | 0 (0%) | 0 (0%) | 0 (0%) | 0 (0%) | 0 (0%) | 0 (0%) | 0 (0%) | 0 (0%) | 0 (0%) | 0 (0%) |
| 1 | 4382 (35.5%) | 5532 (44.8%) | 4760 (38.5%) | 3337 (27.0%) | 2637 (21.4%) | 2587 (20.9%) | 2435 (19.7%) | 2360 (19.1%) | 2368 (19.2%) | 2355 (19.1%) | 2357 (19.1%) |
| 2 | 1824 (14.8%) | 4406 (35.7%) | 5028 (40.7%) | 6065 (49.1%) | 6353 (51.4%) | 6353 (51.4%) | 6288 (50.9%) | 6346 (51.4%) | 6224 (50.4%) | 6209 (50.3%) | 6214 (50.3%) |
| 3 | 506 (4.1%) | 1761 (14.3%) | 1805 (14.6%) | 2036 (16.5%) | 2351 (19.0%) | 2384 (19.3%) | 2557 (20.7%) | 2524 (20.4%) | 2639 (21.4%) | 2617 (21.2%) | 2603 (21.1%) |
| 4+ | 203 (1.6%) | 650 (5.3%) | 756 (6.1%) | 911 (7.4%) | 1008 (8.2%) | 1025 (8.3%) | 1069 (8.7%) | 1119 (9.1%) | 1118 (9.1%) | 1168 (9.5%) | 1175 (9.5%) |
| Missing | 1097 (8.2%) | 1097 (8.2%) | 1097 (8.2%) | 1097 (8.2%) | 1097 (8.2%) | 1097 (8.2%) | 1097 (8.2%) | 1097 (8.2%) | 1097 (8.2%) | 1097 (8.2%) | 1097 (8.2%) |
| **Aid from co-religionists to mothers** | |  |  |  |  |  |  |  |  |  |  |
| No | 10481 (90.7%) | 10481 (90.7%) | 10481 (90.7%) | 10503 (90.9%) | 10470 (90.6%) | 10462 (90.5%) | 10381 (89.8%) | 10399 (90.0%) | 10360 (89.7%) | 10360 (89.7%) | 10360 (89.7%) |
| Yes | 1074 (9.3%) | 1074 (9.3%) | 1074 (9.3%) | 1052 (9.1%) | 1085 (9.4%) | 1093 (9.5%) | 1174 (10.2%) | 1156 (10.0%) | 1195 (10.3%) | 1195 (10.3%) | 1195 (10.3%) |
| Missing | 1891 (14.1%) | 1891 (14.1%) | 1891 (14.1%) | 1891 (14.1%) | 1891 (14.1%) | 1891 (14.1%) | 1891 (14.1%) | 1891 (14.1%) | 1891 (14.1%) | 1891 (14.1%) | 1891 (14.1%) |
| **Partner present** | |  |  |  |  |  |  |  |  |  |  |
| No | 887 (7.1%) | 1231 (9.9%) | 1012 (8.2%) | 946 (7.6%) | 887 (7.1%) | 1001 (8.1%) | 1239 (10.0%) | 1225 (9.9%) | 1209 (9.7%) | 1044 (8.4%) | 1044 (8.4%) |
| Yes | 11522 (92.9%) | 11178 (90.1%) | 11397 (91.8%) | 11463 (92.4%) | 11522 (92.9%) | 11408 (91.9%) | 11170 (90.0%) | 11184 (90.1%) | 11200 (90.3%) | 11365 (91.6%) | 11365 (91.6%) |
| Missing | 1037 (7.7%) | 1037 (7.7%) | 1037 (7.7%) | 1037 (7.7%) | 1037 (7.7%) | 1037 (7.7%) | 1037 (7.7%) | 1037 (7.7%) | 1037 (7.7%) | 1037 (7.7%) | 1037 (7.7%) |
| **Partner’s children in household** | |  |  |  |  |  |  |  |  |  |  |
| 0 | 12186 (98.8%) | 12198 (98.9%) | 12176 (98.7%) | 12168 (98.7%) | 12156 (98.6%) | 12138 (98.4%) | 12114 (98.2%) | 12121 (98.3%) | 12096 (98.1%) | 12100 (98.1%) | 12106 (98.2%) |
| 1 | 95 (0.8%) | 80 (0.6%) | 93 (0.8%) | 99 (0.8%) | 90 (0.7%) | 109 (0.9%) | 130 (1.1%) | 114 (0.9%) | 142 (1.2%) | 123 (1.0%) | 110 (0.9%) |
| 2 | 40 (0.3%) | 48 (0.4%) | 48 (0.4%) | 47 (0.4%) | 64 (0.5%) | 66 (0.5%) | 68 (0.6%) | 70 (0.6%) | 68 (0.6%) | 82 (0.7%) | 87 (0.7%) |
| 3 | 8 (0.1%) | 5 (0.0%) | 10 (0.1%) | 13 (0.1%) | 17 (0.1%) | 14 (0.1%) | 13 (0.1%) | 18 (0.1%) | 18 (0.1%) | 19 (0.2%) | 20 (0.2%) |
| 4+ | 4 (0.0%) | 2 (0.0%) | 6 (0.0%) | 6 (0.0%) | 6 (0.0%) | 6 (0.0%) | 8 (0.1%) | 10 (0.1%) | 9 (0.1%) | 9 (0.1%) | 10 (0.1%) |
| Missing | 1113 (8.3%) | 1113 (8.3%) | 1113 (8.3%) | 1113 (8.3%) | 1113 (8.3%) | 1113 (8.3%) | 1113 (8.3%) | 1113 (8.3%) | 1113 (8.3%) | 1113 (8.3%) | 1113 (8.3%) |
| **Mother's Social network support** | |  |  |  |  |  |  |  |  |  |  |
| Mean (SD) | 23.2 (3.90) | 23.2 (3.77) | 23.1 (4.11) | 22.9 (3.88) | 22.6 (3.84) | 22.3 (4.03) | 22.7 (4.22) | 22.6 (4.11) | 22.5 (4.15) | 22.5 (4.33) | 22.5 (4.33) |
| Missing | 1520 (11.3%) | 1520 (11.3%) | 1520 (11.3%) | 1520 (11.3%) | 1520 (11.3%) | 1520 (11.3%) | 1520 (11.3%) | 1520 (11.3%) | 1520 (11.3%) | 1520 (11.3%) | 1520 (11.3%) |
| **Mother's weekly hours work** | |  |  |  |  |  |  |  |  |  |  |
| Mean (SD) | 3.35 (8.87) | 6.33 (9.34) | 9.30 (12.5) | 10.3 (12.5) | 11.4 (12.2) | 12.4 (13.2) | 12.7 (13.2) | 13.0 (13.5) | 13.1 (13.5) | 13.3 (13.6) | 13.4 (13.8) |
| Missing | 2498 (18.6%) | 2498 (18.6%) | 2498 (18.6%) | 2498 (18.6%) | 2498 (18.6%) | 2498 (18.6%) | 2498 (18.6%) | 2498 (18.6%) | 2498 (18.6%) | 2498 (18.6%) | 2498 (18.6%) |
| **Mother’s age** | |  |  |  |  |  |  |  |  |  |  |
| Mean (SD) | 28.0 (4.94) | 29.0 (4.94) | 30.0 (4.94) | 31.0 (4.94) | 32.0 (4.94) | 33.0 (4.94) | 34.0 (4.94) | 35.0 (4.94) | 36.0 (4.94) | 37.0 (4.94) | 38.0 (4.94) |
| Missing | 754 (5.6%) | 754 (5.6%) | 754 (5.6%) | 754 (5.6%) | 754 (5.6%) | 754 (5.6%) | 754 (5.6%) | 754 (5.6%) | 754 (5.6%) | 754 (5.6%) | 754 (5.6%) |
| **Focal child sex** | |  |  |  |  |  |  |  |  |  |  |
| Female | 6539 (48.9%) | 6539 (48.9%) | 6539 (48.9%) | 6539 (48.9%) | 6539 (48.9%) | 6539 (48.9%) | 6539 (48.9%) | 6539 (48.9%) | 6539 (48.9%) | 6539 (48.9%) | 6539 (48.9%) |
| Male | 6835 (51.1%) | 6835 (51.1%) | 6835 (51.1%) | 6835 (51.1%) | 6835 (51.1%) | 6835 (51.1%) | 6835 (51.1%) | 6835 (51.1%) | 6835 (51.1%) | 6835 (51.1%) | 6835 (51.1%) |
| Missing | 72 (0.5%) | 72 (0.5%) | 72 (0.5%) | 72 (0.5%) | 72 (0.5%) | 72 (0.5%) | 72 (0.5%) | 72 (0.5%) | 72 (0.5%) | 72 (0.5%) | 72 (0.5%) |
| **Focal child ethnicity** | |  |  |  |  |  |  |  |  |  |  |
| Non-white | 528 (4.8%) | 528 (4.8%) | 528 (4.8%) | 528 (4.8%) | 528 (4.8%) | 528 (4.8%) | 528 (4.8%) | 528 (4.8%) | 528 (4.8%) | 528 (4.8%) | 528 (4.8%) |
| White | 10445 (95.2%) | 10445 (95.2%) | 10445 (95.2%) | 10445 (95.2%) | 10445 (95.2%) | 10445 (95.2%) | 10445 (95.2%) | 10445 (95.2%) | 10445 (95.2%) | 10445 (95.2%) | 10445 (95.2%) |
| Missing | 2473 (18.4%) | 2473 (18.4%) | 2473 (18.4%) | 2473 (18.4%) | 2473 (18.4%) | 2473 (18.4%) | 2473 (18.4%) | 2473 (18.4%) | 2473 (18.4%) | 2473 (18.4%) | 2473 (18.4%) |
| **Mother’s weekly household income** | |  |  |  |  |  |  |  |  |  |  |
| <100 GBP | 791 (8.5%) | 791 (8.5%) | 791 (8.5%) | 791 (8.5%) | 724 (7.7%) | 617 (6.6%) | 533 (5.7%) | 583 (6.2%) | 583 (6.2%) | 583 (6.2%) | 583 (6.2%) |
| 100-199 GBP | 1635 (17.5%) | 1635 (17.5%) | 1635 (17.5%) | 1635 (17.5%) | 1501 (16.0%) | 1460 (15.6%) | 1323 (14.1%) | 1278 (13.7%) | 1278 (13.7%) | 1278 (13.7%) | 1278 (13.7%) |
| 200-299 GBP | 2618 (28.0%) | 2618 (28.0%) | 2618 (28.0%) | 2618 (28.0%) | 2398 (25.6%) | 2235 (23.9%) | 1990 (21.3%) | 1894 (20.2%) | 1894 (20.2%) | 1894 (20.2%) | 1894 (20.2%) |
| 300-399 GBP | 1990 (21.3%) | 1990 (21.3%) | 1990 (21.3%) | 1990 (21.3%) | 2127 (22.7%) | 2542 (27.2%) | 2344 (25.1%) | 2026 (21.7%) | 2026 (21.7%) | 2026 (21.7%) | 2026 (21.7%) |
| >400 GBP | 2320 (24.8%) | 2320 (24.8%) | 2320 (24.8%) | 2320 (24.8%) | 2604 (27.8%) | 2500 (26.7%) | 3164 (33.8%) | 3573 (38.2%) | 3573 (38.2%) | 3573 (38.2%) | 3573 (38.2%) |
| Missing | 4092 (30.4%) | 4092 (30.4%) | 4092 (30.4%) | 4092 (30.4%) | 4092 (30.4%) | 4092 (30.4%) | 4092 (30.4%) | 4092 (30.4%) | 4092 (30.4%) | 4092 (30.4%) | 4092 (30.4%) |
| **Mother’s church attendance** | |  |  |  |  |  |  |  |  |  |  |
| Never | 6661 (56.5%) | 6586 (55.9%) | 6429 (54.6%) | 6510 (55.3%) | 6439 (54.7%) | 6473 (54.9%) | 5008 (42.5%) | 4996 (42.4%) | 6062 (51.5%) | 6133 (52.1%) | 6133 (52.1%) |
| Yearly | 3415 (29.0%) | 3490 (29.6%) | 3577 (30.4%) | 3242 (27.5%) | 3230 (27.4%) | 3186 (27.0%) | 4597 (39.0%) | 4569 (38.8%) | 3618 (30.7%) | 3544 (30.1%) | 3544 (30.1%) |
| Monthly | 822 (7.0%) | 852 (7.2%) | 967 (8.2%) | 1191 (10.1%) | 1149 (9.8%) | 1076 (9.1%) | 1106 (9.4%) | 1188 (10.1%) | 1069 (9.1%) | 977 (8.3%) | 977 (8.3%) |
| Weekly | 883 (7.5%) | 853 (7.2%) | 808 (6.9%) | 838 (7.1%) | 963 (8.2%) | 1046 (8.9%) | 1070 (9.1%) | 1028 (8.7%) | 1032 (8.8%) | 1127 (9.6%) | 1127 (9.6%) |
| Missing | 1665 (12.4%) | 1665 (12.4%) | 1665 (12.4%) | 1665 (12.4%) | 1665 (12.4%) | 1665 (12.4%) | 1665 (12.4%) | 1665 (12.4%) | 1665 (12.4%) | 1665 (12.4%) | 1665 (12.4%) |
| **Mother’s education** | |  |  |  |  |  |  |  |  |  |  |
| CSE/none | 1541 (14.5%) | 1541 (14.5%) | 1541 (14.5%) | 1541 (14.5%) | 1541 (14.5%) | 1541 (14.5%) | 1541 (14.5%) | 1541 (14.5%) | 1541 (14.5%) | 1541 (14.5%) | 1541 (14.5%) |
| Vocational | 1103 (10.4%) | 1103 (10.4%) | 1103 (10.4%) | 1103 (10.4%) | 1103 (10.4%) | 1103 (10.4%) | 1103 (10.4%) | 1103 (10.4%) | 1103 (10.4%) | 1103 (10.4%) | 1103 (10.4%) |
| O level | 3943 (37.2%) | 3943 (37.2%) | 3943 (37.2%) | 3943 (37.2%) | 3943 (37.2%) | 3943 (37.2%) | 3943 (37.2%) | 3943 (37.2%) | 3943 (37.2%) | 3943 (37.2%) | 3943 (37.2%) |
| A level | 2550 (24.1%) | 2550 (24.1%) | 2550 (24.1%) | 2550 (24.1%) | 2550 (24.1%) | 2550 (24.1%) | 2550 (24.1%) | 2550 (24.1%) | 2550 (24.1%) | 2550 (24.1%) | 2550 (24.1%) |
| College degree | 1455 (13.7%) | 1455 (13.7%) | 1455 (13.7%) | 1455 (13.7%) | 1455 (13.7%) | 1455 (13.7%) | 1455 (13.7%) | 1455 (13.7%) | 1455 (13.7%) | 1455 (13.7%) | 1455 (13.7%) |
| Missing | 2854 (21.2%) | 2854 (21.2%) | 2854 (21.2%) | 2854 (21.2%) | 2854 (21.2%) | 2854 (21.2%) | 2854 (21.2%) | 2854 (21.2%) | 2854 (21.2%) | 2854 (21.2%) | 2854 (21.2%) |

| **Table S3: Descriptive statistics of all variables used in height analyses after imputation.** The total number of mothers included in this dataset is 8,976 and there are a total of 499,709 non-missing data points for the variables in the table below. After exclusions due to missing data in preditor variables, there were 6,561 mothers included in the dataset and 413,182 non-missing data points (as reported Table S6). Percentages indicate percentage of valid cases, except for the missing cases, where the percentages are for all cases. | | | | | | | | | | | | | | |
| --- | --- | --- | --- | --- | --- | --- | --- | --- | --- | --- | --- | --- | --- | --- |
| Year | **4 months** | **8 months** | **1 year** | **1 year, 6 months** | **2 years, 1 month** | **2 years, 7 months** | **3 years, 1 month** | **3 years, 7 months** | **4 years, 1 month** | **5 years, 1 month** | **7 years** | **8 years** | **9 years** | **10 years** |
| **Focal child height (cm)** | |  |  |  |  |  |  |  |  |  |  |  |  |  |
| Mean (SD) | 62.6 (2.26) | 70.2 (2.41) | 75.6 (2.54) | 81.8 (2.84) | 86.9 (3.06) | 91.6 (3.34) | 95.6 (3.53) | 99.3 (3.74) | 103 (3.97) | 110 (4.37) | 126 (5.66) | 133 (5.85) | 140 (6.33) | 144 (6.74) |
| **Mother’s height (cm)** | |  |  |  |  |  |  |  |  |  |  |  |  |  |
| Mean (SD) | 165 (6.41) | 164  (6.49) | 164 (6.44) | 164 (6.49) | 164 (6.45) | 164 (6.44) | 164 (6.48) | 164  (6.46) | 165 (6.49) | 164 (6.25) | 164 (6.62) | 164 (6.62) | 164 (6.61) | 164 (6.61) |
| Missing | 66 (7.3%) | 71  (6.0%) | 69 (6.2%) | 64 (6.1%) | 50 (5.3%) | 55 (5.6%) | 47 (5.0%) | 44  (4.7%) | 41 (4.4%) | 41 (4.6%) | 787 (10.6%) | 586 (9.0%) | 717 (10.3%) | 673 (9.9%) |
| **Focal child number of siblings** | |  |  |  |  |  |  |  |  |  |  |  |  |  |
| 0 | 406 (46.1%) | 549 (46.6%) | 535 (48.2%) | 433 (41.6%) | 386 (41.3%) | 267 (27.4%) | 236 (24.9%) | 155 (16.4%) | 148 (16.1%) | 133 (15.0%) | 941 (13.3%) | 809 (13.0%) | 820 (12.5%) | 811 (12.6%) |
| 1 | 299 (34.0%) | 405 (34.4%) | 339 (30.5%) | 391 (37.6%) | 323 (34.5%) | 469 (48.1%) | 434 (45.9%) | 517 (54.8%) | 503 (54.7%) | 463 (52.3%) | 3917 (55.5%) | 3497 (56.1%) | 3691 (56.1%) | 3632 (56.2%) |
| 2 | 128 (14.5%) | 163 (13.8%) | 177 (15.9%) | 150 (14.4%) | 170 (18.2%) | 165 (16.9%) | 207 (21.9%) | 194 (20.6%) | 194 (21.1%) | 218 (24.6%) | 1624 (23.0%) | 1412 (22.6%) | 1483 (22.5%) | 1449 (22.4%) |
| 3 | 36 (4.1%) | 46  (3.9%) | 41 (3.7%) | 46 (4.4%) | 37 (4.0%) | 53 (5.4%) | 46 (4.9%) | 60  (6.4%) | 58 (6.3%) | 54 (6.1%) | 439 (6.2%) | 390 (6.3%) | 440 (6.7%) | 425 (6.6%) |
| 4+ | 11 (1.3%) | 14  (1.2%) | 18 (1.6%) | 20 (1.9%) | 19 (2.0%) | 21 (2.2%) | 23 (2.4%) | 18  (1.9%) | 16 (1.7%) | 17 (1.9%) | 132 (1.9%) | 126 (2.0%) | 150 (2.3%) | 140 (2.2%) |
| Missing | 22 (2.4%) | 8  (0.7%) | 6  (0.5%) | 5  (0.5%) | 6  (0.6%) | 3  (0.3%) | 3  (0.3%) | 2  (0.2%) | 3  (0.3%) | 3  (0.3%) | 404 (5.4%) | 264 (4.1%) | 347 (5.0%) | 332 (4.9%) |
| **Mother’s aid from co-religionists** | |  |  |  |  |  |  |  |  |  |  |  |  |  |
| 0 | 755 (87.5%) | 1012 (88.1%) | 957 (88.3%) | 901 (88.4%) | 812 (88.5%) | 848 (88.8%) | 821 (88.3%) | 822 (88.4%) | 803 (88.7%) | 768 (88.1%) | 6289 (88.3%) | 5525 (87.8%) | 5817 (87.5%) | 5715 (87.7%) |
| 1 | 108 (12.5%) | 137 (11.9%) | 127 (11.7%) | 118 (11.6%) | 106 (11.5%) | 107 (11.2%) | 109 (11.7%) | 108 (11.6%) | 102 (11.3%) | 104 (11.9%) | 831 (11.7%) | 771 (12.2%) | 828 (12.5%) | 800 (12.3%) |
| Missing | 39 (4.3%) | 36  (3.0%) | 32 (2.9%) | 26 (2.5%) | 23 (2.4%) | 23 (2.4%) | 19 (2.0%) | 16  (1.7%) | 17 (1.8%) | 16 (1.8%) | 337 (4.5%) | 202 (3.1%) | 286 (4.1%) | 274 (4.0%) |
| **Mother’s education** | |  |  |  |  |  |  |  |  |  |  |  |  |  |
| CSE/none | 81 (9.8%) | 81  (7.5%) | 98 (9.4%) | 92 (9.4%) | 78 (8.8%) | 81 (8.8%) | 73 (8.2%) | 76  (8.5%) | 73 (8.4%) | 71 (8.5%) | 639 (9.8%) | 564 (9.7%) | 616 (10.1%) | 601 (10.0%) |
| Vocational | 79 (9.5%) | 111 (10.3%) | 105 (10.1%) | 103 (10.6%) | 98 (11.0%) | 94 (10.3%) | 93 (10.5%) | 90  (10.1%) | 89 (10.2%) | 80 (9.6%) | 580 (8.9%) | 505 (8.7%) | 524 (8.6%) | 524 (8.8%) |
| O level | 310 (37.3%) | 413 (38.5%) | 395 (37.9%) | 364 (37.3%) | 333 (37.5%) | 3339 (37.0%) | 333 (37.5%) | 335 (37.7%) | 328 (37.6%) | 315 (37.7%) | 2433 (37.2%) | 2137 (36.9%) | 2246 (36.9%) | 2219 (37.1%) |
| A level | 222 (26.7%) | 297 (27.7%) | 282 (27.1%) | 267 (27.4%) | 245 (27.6%) | 261 (28.5%) | 249 (28.0%) | 247 (27.8%) | 243 (27.9%) | 239 (28.6%) | 1793 (27.4%) | 1603 (28.3%) | 1695 (27.8%) | 1655 (27.6%) |
| College degree | 138 (16.6%) | 172 (16.0%) | 161 (15.5%) | 150 (15.4%) | 134 (15.1%) | 141 (15.4%) | 140 (15.8%) | 141 (15.9%) | 139 (15.9%) | 131 (15.7%) | 1091 (16.7%) | 987 (17.0%) | 1006 (16.5%) | 988 (16.5%) |
| Missing | 72 (8.0%) | 91  (7.7%) | 75 (6.7%) | 69 (6.6%) | 53 (5.6%) | 62 (6.3%) | 61 (6.4%) | 57  (6.0%) | 50 (5.4%) | 52 (5.9%) | 921 (12.4%) | 702 (10.8%) | 844 (12.2%) | 802 (11.8%) |
| **Mother’s partner present** | |  |  |  |  |  |  |  |  |  |  |  |  |  |
| No | 43 (4.8%) | 53  (4.5%) | 73 (6.6%) | 68 (6.5%) | 68 (7.2%) | 50 (5.1%) | 46 (4.9%) | 46  (4.9%) | 46 (5.0%) | 84 (9.5%) | 612 (8.5%) | 514 (8.1%) | 433 (6.4%) | 412 (6.2%) |
| Yes | 846 (95.2%) | 1124 (95.5%) | 1036 (93.4%) | 972 (93.5%) | 870 (92.8%) | 927 (94.9%) | 902 (95.1%) | 899 (95.1%) | 875 (95.0%) | 803 (90.5%) | 6608 (91.5%) | 5859 (91.9%) | 6320 (93.6%) | 6207 (93.8%) |
| Missing | 13 (1.4%) | 8  (0.7%) | 7  (0.6%) | 5  (0.5%) | 3  (0.3%) | 1  (0.1%) | 1  (0.1%) | 1  (0.1%) | 1  (0.1%) | 1  (0.1%) | 237 (3.2%) | 125 (1.9%) | 178 (2.6%) | 170 (2.5%) |
| **Mother’s partner’s children in household** | |  |  |  |  |  |  |  |  |  |  |  |  |  |
| 0 | 880 (99.0%) | 1160 (98.6%) | 1095 (98.6%) | 1029 (99.0%) | 922 (98.7%) | 971 (99.6%) | 930 (98.3%) | 932 (98.7%) | 908 (98.8%) | 870 (98.4%) | 6899 (98.1%) | 6103 (98.1%) | 6440 (98.1%) | 6325 (98.2%) |
| 1 | 5  (0.6%) | 12  (1.0%) | 10  (0.9%) | 5  (0.5%) | 7  (0.7%) | 3  (0.3%) | 10  (1.1%) | 4  (0.4%) | 3  (0.3%) | 6  (0.7%) | 83  (1.2%) | 81  (1.3%) | 76  (1.2%) | 62  (1.0%) |
| 2 | 1  (0.1%) | 2  (0.2%) | 3  (0.3%) | 2  (0.2%) | 4  (0.4%) | 1  (0.1%) | 5  (0.5%) | 5  (0.5%) | 6  (0.7%) | 4  (0.5%) | 41  (0.6%) | 26  (0.4%) | 42  (0.6%) | 43  (0.7%) |
| 3 | 2  (0.2%) | 2  (0.2%) | 2  (0.2%) | 3  (0.3%) | 1  (0.1%) | 0  (0.0%) | 1  (0.1%) | 3  (0.3%) | 2  (0.2%) | 4  (0.5%) | 6  (0.1%) | 8  (0.1%) | 6  (0.1%) | 8  (0.1%) |
| 4+ | 1  (0.1%) | 0  (0.0%) | 0  (0.0%) | 0  (0.0%) | 0  (0.0%) | 0  (0.0%) | 0  (0.0%) | 0  (0.0%) | 0  (0.0%) | 0  (0.0%) | 5  (0.1%) | 4  (0.1%) | 4  (0.1%) | 4  (0.1%) |
| Missing | 13 (1.4%) | 9  (0.8%) | 6  (0.5%) | 6  (0.6%) | 7  (0.7%) | 3  (0.3%) | 3  (0.3%) | 2  (0.2%) | 3  (0.3%) | 4  (0.5%) | 423 (5.7%) | 276 (4.2%) | 363 (5.2%) | 347 (5.1%) |
| **Mother’s social network support** | |  |  |  |  |  |  |  |  |  |  |  |  |  |
| Mean (SD) | 23.6 (3.82) | 23.7 (3.58) | 23.7 (3.60) | 23.6 (3.98) | 23.4 (3.81) | 23.3 (3.63) | 23.2 (3.56) | 23.1 (3.47) | 22.9 (3.51) | 22.8 (3.65) | 22.9 (3.93) | 22.8 (3.94) | 22.8 (4.20) | 22.7 (4.19) |
| Missing | 24 (2.7%) | 24  (2.0%) | 23 (2.1%) | 18 (1.7%) | 13 (1.4%) | 12 (1.2%) | 12 (1.3%) | 7  (0.7%) | 8  (0.9%) | 7  (0.8%) | 248 (3.3%) | 147 (2.3%) | 213 (3.1%) | 203 (3.0%) |
| **Mother's weekly hours work** | |  |  |  |  |  |  |  |  |  |  |  |  |  |
| Mean (SD) | 2.73 (7.58) | 5.38 (7.94) | 7.74 (9.58) | 10.6 (12.5) | 11.0 (11.9) | 11.3 (12.6) | 12.1 (12.0) | 13.0 (11.9) | 14.0 (12.4) | 14.3 (13.0) | 15.0 (13.5) | 15.3 (13.5) | 15.4 (13.5) | 15.5 (13.7) |
| Missing | 42 (4.7%) | 45  (3.8%) | 41 (3.7%) | 35 (3.3%) | 25 (2.7%) | 24 (2.5%) | 22 (2.3%) | 16  (1.7%) | 18 (2.0%) | 22 (2.5%) | 615 (8.2%) | 431 (6.6%) | 548 (7.9%) | 517 (7.6%) |
| **Mother’s age at birth** | |  |  |  |  |  |  |  |  |  |  |  |  |  |
| Mean (SD) | 29.0 (4.66) | 30.1 (4.51) | 30.1 (4.46) | 30.6 (4.49) | 31.2 (4.39) | 32.2 (4.46) | 32.3 (4.38) | 33.3 (4.37) | 33.3 (4.40) | 34.3 (4.41) | 36.0 (4.55) | 37.1 (4.50) | 38.0 (4.52) | 39.1 (4.50) |
| Missing | 0 (0%) | 0 (0%) | 0 (0%) | 0 (0%) | 0 (0%) | 0 (0%) | 0 (0%) | 0 (0%) | 0 (0%) | 0 (0%) | 436 (5.8%) | 295 (4.5%) | 389 (5.6%) | 369 (5.4%) |
| **Focal child sex** | |  |  |  |  |  |  |  |  |  |  |  |  |  |
| Female | 416 (46.1%) | 542 (45.7%) | 516 (46.2%) | 477 (45.6%) | 427 (45.4%) | 446 (45.6%) | 435 (45.8%) | 424 (44.8%) | 414  (44.9%) | 401 (45.2%) | 3691 (49.5%) | 3264 (50.3%) | 3532 (51.0%) | 3456 (50.9%) |
| Male | 486 (53.9%) | 643 (54.3%) | 600 (53.8%) | 568 (54.4%) | 514 (54.6%) | 532 (54.4%) | 514 (54.2%) | 522 (55.2%) | 508 (55.1%) | 487 (54.8%) | 3762 (50.5%) | 3230 (49.7%) | 3395 (49.0%) | 3330 (49.1%) |
| Missing | 0 (0%) | 0 (0%) | 0 (0%) | 0 (0%) | 0 (0%) | 0 (0%) | 0 (0%) | 0 (0%) | 0 (0%) | 0 (0%) | 4 (0.1%) | 4 (0.1%) | 4 (0.1%) | 3 (0.0%) |
| **Focal child ethnicity** | |  |  |  |  |  |  |  |  |  |  |  |  |  |
| Non-white | 29 (3.4%) | 35  (3.1%) | 32 (3.0%) | 31 (3.1%) | 26 (2.9%) | 29 (3.1%) | 26 (2.8%) | 24  (2.6%) | 26 (2.9%) | 24 (2.8%) | 257 (3.9%) | 214 (3.6%) | 233 (3.8%) | 226 (3.7%) |
| White | 822 (96.6%) | 1095 (96.9%) | 1041 (97.0%) | 974 (96.9%) | 881 (97.1%) | 914 (96.9%) | 887 (97.2%) | 889 (97.4%) | 866 (97.1%) | 830 (97.2%) | 6405 (96.1%) | 5683 (96.4%) | 5967 (96.2%) | 5874 (96.3%) |
| Missing | 51 (5.7%) | 55  (4.6%) | 43 (3.9%) | 40 (3.8%) | 34 (3.6%) | 35 (3.6%) | 36 (3.8%) | 33  (3.5%) | 30 (3.3%) | 34 (3.8%) | 795 (10.7%) | 601 (9.2%) | 731 (10.5%) | 689 (10.1%) |
| **Mother’s weekly household income** | |  |  |  |  |  |  |  |  |  |  |  |  |  |
| <100 GBP | 41 (5.4%) | 41  (4.2%) | 62 (6.3%) | 55 (5.9%) | 51 (5.9%) | 57 (6.4%) | 31 (3.5%) | 41  (4.7%) | 21 (3.6%) | 22 (2.7%) | 236 (3.7%) | 197 (3.4%) | 235 (3.9%) | 221 (3.7%) |
| 100-199 GBP | 126 (16.5%) | 126 (12.9%) | 159 (16.2%) | 142 (15.1%) | 142 (16.5%) | 145 (16.3%) | 174 (19.8%) | 119 (13.6%) | 125 (14.5%) | 123 (14.9%) | 639 (10.8%) | 594 (10.4%) | 649 (10.8%) | 631 (10.7%) |
| 200-299 GBP | 206 (27.0%) | 269 (27.6%) | 256 (26.1%) | 250 (26.6%) | 218 (25.3%) | 222 (25.0%) | 143 (16.2%) | 202 (23.0%) | 171 (19.8%) | 116 (14.0%) | 1261 (19.6%) | 1108 (19.4%) | 1147 (19.1%) | 1128 (19.1%) |
| 300-399 GBP | 159 (20.8%) | 227 (23.3%) | 211 (21.5%) | 205 (21.8%) | 192 (22.3%) | 194 (21.8%) | 305 (34.6%) | 232 (26.4%) | 268 (31.0%) | 322 (39.0%) | 1464 (22.7%) | 1303 (22.8%) | 1375 (22.9%) | 1363 (23.1%) |
| >400 GBP | 231 (30.3%) | 310 (31.9%) | 292 (29.8%) | 288 (30.6%) | 259 (30.0%) | 271 (30.5%) | 228 (25.9%) | 284 (32.3%) | 270 (31.2%) | 243 (29.4%) | 2786 (43.3%) | 2517 (44.0%) | 2601 (43.3%) | 2554 (43.3%) |
| Missing | 139 (15.4%) | 153 (12.9%) | 136 (12.2%) | 105 (10.0%) | 79 (8.4%) | 89 (9.1%) | 68 (7.2%) | 68  (7.2%) | 57 (6.2%) | 62 (7.0%) | 1017 (13.6%) | 779 (12.0%) | 924 (13.3%) | 892 (13.1%) |
| **Mother’s church attendance** | |  |  |  |  |  |  |  |  |  |  |  |  |  |
| Never | 455 (52.6%) | 598 (51.9%) | 547 (50.5%) | 497 (48.6%) | 444 (48.4%) | 486 (50.8%) | 461 (49.6%) | 463 (49.7%) | 447 (49.3%) | 432 (49.5%) | 2395 (33.3%) | 2867 (45.2%) | 3089 (46.1%) | 3018 (46.0%) |
| Yearly | 241 (27.9%) | 342 (29.7%) | 336 (31.0%) | 330 (32.3%) | 298 (32.5%) | 259 (27.1%) | 261 (28.1%) | 260 (27.9%) | 253 (27.9%) | 237 (27.1%) | 3179 (44.3%) | 2108 (33.2%) | 2179 (32.5%) | 2152 (32.8%) |
| Monthly | 85 (9.8%) | 104 (9.0%) | 106 (9.8%) | 111 (10.9%) | 103 (11.2%) | 128 (13.4%) | 125 (13.4%) | 111 (11.9%) | 107 (11.8%) | 103 (11.8%) | 870 (12.1%) | 658 (11.0%) | 637 (9.5%) | 628 (9.6%) |
| Weekly | 84 (9.7%) | 108 (9.4%) | 95 (8.8%) | 84 (8.2%) | 73 (8.0%) | 83 (8.7%) | 83 (8.9%) | 97 (10.4%) | 100 (11.0%) | 101 (11.6%) | 740 (10.3%) | 669 (10.5%) | 795 (11.9%) | 767 (11.7%) |
| Missing | 37 (4.1%) | 33  (2.8%) | 32 (2.9%) | 23 (2.2%) | 23 (2.4%) | 22 (2.2%) | 19 (2.0%) | 15  (1.6%) | 15 (1.6%) | 15 (1.7%) | 273 (3.7%) | 156 (2.4%) | 231 (3.3%) | 224 (3.3%) |

**Table S4: Results of Bayesian regression models predicting mothers’ social network support (Model 1; n = 8,209) and aid from co-religionists (Model 2; n = 8,209) from birth to 10 years of age.**

|  | Model 1 | Model 2 |
| --- | --- | --- |
|  | **Regression Estimate**  **(with 95% CI)** | **Regression Estimate**  **(with 95% CI)** |
| Intercept | 22.60(22.09, 23.07) | -9.23 (-10.09, -8.38) |
| Year | -0.10 (-0.15, -0.05) | 0.02 (-0.05, 0.08) |
| Mother's church attendance | |  |
| Yearly | 0.15 (0.11, 0.20) | 2.21 (1.96, 2.47) |
| Monthly | 0.18 (0.11, 0.26) | 6.07 (5.77, 6.36) |
| Weekly | 0.23 (0.02, 0.34) | 8.88 (8.56, 9.21) |
| Mother’s social network support (centered) | NA | 0.44 (0.34, 0.54) |
| Mother’s aid from co-religionists | 0.37 (0.29, 0.46) | NA |
| Mother’s weekly hours of work (centered) | 0.00 (0.00, 0.00) | -0.01 (-0.02, 0.00) |
| Mother’s age (centered) | -0.07 (-0.09, -0.06) | 0.10 (0.07, 0.14) |
| Mother’s education (centered) | 0.51 (0.44, 0.58) | 0.60 (0.45, 0.74) |
| Mother’s household income (centered) | 0.10 (0.07, 0.13) | -0.13 (-0.22, -0.04) |
| Male focal child | -0.10 (-0.24, 0.05) | 0.21 (-0.07, 0.50) |
| Mother’s partner present | -0.30 (-0.38, -0.22) | 0.32 (0.10, 0.64) |
| Focal child ethnicity (1=white, 0=non-white) | 1.11 (0.73, 1.49) | -1.48 (-2.15, -0.79) |

| Table S5: Results of Bayesian regression models predicting total offspring from birth to 10 years of age (n = 8,207). Models 2 and 3 include interaction  terms of frequency of mother’s ritual attendance and mother’s social network support (Model 2) and frequency of mother’s ritual attendance and  mother’s aid from co-religionists (Model 2). | | | |
| --- | --- | --- | --- |
|  | **Model 1**  **Regression Estimate**  **(with 95% CI)** | **Model 2**  **Regression Estimate**  **(with 95% CI)** | **Model 3**  **Regression Estimate**  **(with 95% CI)** |
| Intercept | 0.16 (-0.18, 0.51) | 0.16 (-0.20, 0.50) | 0.16 (-0.20, 0.54) |
| Year | 0.07 (0.01, 0.13) | 0.07 (0.01, 0.13) | 0.07 (0.01, 0.13) |
| Mother’s partner’s children in household (centered) | 0.07 (0.04, 0.09) | 0.07 (0.04, 0.09) | 0.07 (0.04, 0.09) |
| Mother's church attendance | |  |  |
| Yearly | 0.01 (-0.01, 0.02) | 0.00 (-0.01, 0.02) | 0.00 (-0.01, 0.02) |
| Monthly | 0.04 (0.01, 0.06) | 0.04 (0.01, 0.06) | 0.04 (0.01, 0.06) |
| Weekly | 0.07 (0.04, 0.10) | 0.07 (0.04, 0.10) | 0.06 (0.02, 0.10) |
| Mother’s social network support (scaled) | -0.02 (-0.03, -0.01) | -0.02 (-0.03, -0.01) | -0.02 (-0.03, -0.01) |
| Mother’s aid from co-religionists | 0.04 (0.02, 0.07) | 0.04 (0.02, 0.07) | 0.03 (-0.07, 0.12) |
| Mother’s weekly hours of work (centered) | 0.00 (0.00, 0.00) | 0.00 (0.00, 0.00) | 0.00 (0.00, 0.00) |
| Mother’s age (centered) | 0.02 (0.02, 0.02) | 0.02 (0.02, 0.02) | 0.02 (0.02, 0.02) |
| Mother’s education (centered) | -0.04 (-0.05, -0.03) | -0.04 (-0.05, -0.03) | -0.04 (-0.05, -0.03) |
| Mother’s household income (centered) | 0.00 (-0.01, 0.01) | 0.00 (-0.01, 0.01) | 0.00 (-0.01, 0.01) |
| Focal child sex (1=male, 0=female) | 0.01 (-0.01, 0.02) | 0.01 (-0.01, 0.02) | 0.01 (-0.01, 0.02) |
| Mother’s partner present | 0.07 (0.04, 0.09) | 0.07 (0.04, 0.09) | 0.07 (0.04, 0.09) |
| Focal child ethnicity (1=white, 0=non-white) | 0.05 (0.00, 0.09) | 0.05 (0.00, 0.09) | 0.05 (0.00, 0.09) |
|  |  |  |  |
| Yearly church attendance * social network support | NA | 0.00 (-0.01, 0.02) | NA |
| Monthly church attendance * social network support | NA | 0.00 (-0.02, 0.02) | NA |
| Weekly church attendance * social network support | NA | 0.00 (-0.02, 0.03) | NA |
|  |  |  |  |
| Yearly church attendance * aid from co-religionists | NA | NA | 0.02 (-0.08, 0.13) |
| Monthly church attendance * aid from co-religionists | NA | NA | 0.01 (-0.09, 0.11) |
| Weekly church attendance * aid from co-religionists | NA | NA | 0.02 (-0.08, 0.13) |

| Table S6: Results of longitudinal Bayesian regression models predicting focal child height from birth to 10 years of age (n=6,561). Models 2 and 3  include interaction terms of frequency of mother’s ritual attendance and mother’s social network support (Model 2) and frequency of mother’s  ritual attendance and mother’s aid from co-religionists (Model 2). | | | |
| --- | --- | --- | --- |
|  | **Model 1**  **Regression Estimate**  **(with 95% CI)** | **Model 2**  **Regression Estimate**  **(with 95% CI)** | **Model 3**  **Regression Estimate**  **(with 95% CI)** |
| Intercept | 68.38 (64.67, 72.01) | 68.37 (64.89, 71.83) | 68.34 (64.78, 71.82) |
| Year | 8.06 (7.38, 8.77) | 8.05 (7.39, 8.69) | 8.06 (7.38, 8.76) |
| Focal child sibling number (centered) | -0.24 (-0.32, -0.16) | -0.24 (-0.32, -0.16) | -0.24 (-0.32, -0.16) |
| Mother’s partner’s children in household (centered) | 0.10 (-0.11, 0.31) | 0.10 (-0.11, 0.31) | 0.10 (-0.11, 0.31) |
| Mother's church attendance | |  |  |
| Yearly | 0.11 (0.01, 0.21) | 0.11 (0.01, 0.21) | 0.10 (0.00, 0.20) |
| Monthly | 0.16 (0.00, 0.32) | 0.17 (0.01, 0.33) | 0.16 (-0.01, 0.32) |
| Weekly | 0.19 (-0.03, 0.42) | 0.19 (-0.03, 0.42) | 0.15 (-0.11, 0.41) |
| Mother’s social network support (scaled) | -0.01 (-0.08, 0.05) | -0.01 (-0.10, 0.07) | -0.01 (-0.08, 0.05) |
| Mother’s aid from co-religionists | -0.14 (-0.31, 0.03) | -0.14 (-0.31, 0.04) | -0.86 (-1.65, 0.08) |
| Mother’s weekly hours of work (centered) | 0.01 (0.01, 0.02) | 0.01 (0.01, 0.02) | 0.01 (0.01, 0.02) |
| Mother’s age (centered) | 0.06 (0.03, 0.09) | 0.06 (0.03, 0.09) | 0.06 (0.03, 0.09) |
| Mother’s education (centered) | -0.07 (-0.19, 0.05) | -0.07 (-0.18, 0.05) | -0.07 (-0.18, 0.05) |
| Mother’s household income (centered) | -0.08 (-0.15, -0.01) | -0.08 (-0.16, -0.01) | -0.08 (-0.15, -0.01) |
| Focal child sex (1=male, 0=female) | 0.71 (0.47, 0.96) | 0.71 (0.46, 0.98) | 0.71 (0.47, 0.95) |
| Mother’s partner present | 0.18 (-0.02, 0.38) | 0.18 (-0.02, 0.38) | 0.18 (-0.02, 0.39) |
| Focal child ethnicity (1=white, 0=non-white) | -0.92 (-1.59, -0.23) | -0.88 (-1.55, -0.23) | -0.93 (-1.60, -0.29) |
| Mother’s height (centered) | 0.32 (0.30, 0.34) | 0.32 (0.31, 0.34) | 0.32 (0.30, 0.34) |
|  |  |  |  |
| Yearly church attendance * social network support | NA | 0.01 (-0.08, 0.11) | NA |
| Monthly church attendance * social network support | NA | -0.04 (-0.18, 0.10) | NA |
| Weekly church attendance * social network support | NA | -0.01 (-0.18, 0.17) | NA |
|  |  |  |  |
| Yearly church attendance * aid from co-religionists | NA | NA | 0.80 (-0.01, 1.61) |
| Monthly church attendance * aid from co-religionists | NA | NA | 0.71 (-0.10, 1.52) |
| Weekly church attendance * aid from co-religionists | NA | NA | 0.78 (-0.06, 1.62) |

| Table S7: Results of Bayesian regression models predicting focal child’s performance on the Entry Level Assessment (Model 1; n = 1,766), the Stage 1 Assessment (Model 2; n = 5,814), and the WISC-III (Model 3; n = 5,379). | | | |
| --- | --- | --- | --- |
|  | **Model 1**  **Regression Estimate**  **(with 95% CI)** | **Model 2**  **Regression Estimate**  **(with 95% CI)** | **Model 3**  **Regression Estimate**  **(with 95% CI)** |
| Intercept | 27.66 (25.911, 29.39) | 10.58 (10.04, 11.12) | 105.25 (102.61, 107.92) |
| Focal child sibling number (centered) | -0.33 (-0.65, -0.01) | -0.34 (-0.44, -0.25) | -1.34 (-1.81, -0.88) |
| Mother’s partner’s children in household (centered) | 0.83 (-0.64, 2.30) | 0.12 (-0.56, 0.39) | 0.31 (-1.46, 2.16) |
| Mother's church attendance | |  |  |
| Yearly | 0.50 (-0.14, 1.13) | 0.18 (-0.01, 0.37) | -0.16 (-1.12, 0.81) |
| Monthly | 0.11 (-0.84, 1.11) | 0.17 (-0.13, 0.48) | 0.36 (-1.06, 1.79) |
| Weekly | 1.00 (-0.22, 2.24) | 0.36 (-0.02, 0.76) | -0.72 (-2.55, 1.08) |
| Mother’s social network support (scaled) | 0.14 (-0.14, 0.42) | 0.19 (0.10, 0.28) | 0.63 (0.19, 1.07) |
| Mother’s aid from co-religionists | 0.42 (-0.75, 1.61) | 0.11 (-0.26, 0.47) | 2.33 (0.72, 4.01) |
| Mother’s weekly hours of work (centered) | -0.08 (-0.29, 0.13) | -0.14 (-0.20, -0.08) | -0.68 (-0.98, -0.38) |
| Mother’s age (centered) | 0.03 (-0.04, 0.09) | 0.04 (0.02, 0.06) | 0.28 (0.18, 0.38) |
| Mother’s education (centered) | 0.71 (0.45, 0.96) | 0.66 (0.57, 0.74) | 3.95 (3.55, 4.35) |
| Mother’s household income (centered) | 0.80 (0.52, 1.07) | 0.50 (0.41, 0.58) | 1.95 (1.52, 2.38) |
| Focal child sex (1=male, 0=female) | -2.30 (-2.82, -1.77) | -0.99 (-1.15, -0.82) | 0.17 (-0.64, 0.99) |
| Mother’s partner present | -0.46 (-1.69, 0.74) | -0.20 (-0.52, 0.11) | -2.11 (-3.79, -0.44) |
| Focal child ethnicity (1=white, 0=non-white) | 0.83 (-0.64, 2.30) | -0.09 (-0.56, 0.39) | -0.01 (-2.27, 2.19) |

| Table S8: Results of Bayesian regression models predicting focal child’s performance on Entry Assessments (Models 1 and 2; n = 1,766). Models include interaction terms of frequency of mother’s ritual attendance and mother’s social network support (Model 1) and frequency of mother’s ritual attendance and mother’s aid from co-religionists (Model 2). | | |
| --- | --- | --- |
|  | **Model 1**  **Regression Estimate**  **(with 95% CI)** | **Model 2**  **Regression Estimate**  **(with 95% CI)** |
| Intercept | 27.72 (25.95, 29.50) | 27.61 (25.84, 29.40) |
| Focal child sbling number (centered) | -0.31 (-0.64, -0.02) | -0.34 (-0.65, -0.03) |
| Mother’s partner’s children in household (centered) | 0.77 (-0.52, 2.02) | 0.87 (-0.40, 2.11) |
| Mother's church attendance | |  |
| Yearly | 0.52 (-0.13, 1.15) | 0.47 (-0.17, 1.11) |
| Monthly | 0.20 (-0.80, 1.19) | 0.31 (-0.73, 1.37) |
| Weekly | 1.00 (-0.29, 2.23) | 0.79 (-0.76, 2.29) |
| Mother’s social network support (scaled) | 0.38 (0.02, 0.73) | 0.14 (-0.14, 0.42) |
| Mother’s aid from co-religionists | 0.50 (-0.76, 1.74) | -0.58 (-6.24, 5.00) |
| Mother’s weekly hours of work (centered) | -0.09 (-0.30, 0.12) | -0.08 (-0.29, 0.13) |
| Mother’s age (centered) | 0.03 (-0.03, 0.09) | 0.03 (-0.03, 0.09) |
| Mother’s education (centered) | 0.71 (0.46, 0.97) | 0.70 (0.45, 0.96) |
| Mother’s household income (centered) | 0.80 (0.52, 1.07) | 0.80 (0.53, 1.06) |
| Focal child sex (1=male, 0=female) | -2.32 (-2.85, -1.78) | -2.32 (-2.85, -1.78) |
| Mother’s partner present | -0.46 (-1.65, 0.76) | -0.46 (-1.70, 0.78) |
| Focal child ethnicity (1=white, 0=non-white) | 0.81 (-0.66, 2.30) | 0.89 (-0.64, 2.38) |
|  |  |  |
| Yearly church attendance * social network support | -0.54 (-1.21, 0.12) | NA |
| Monthly church attendance * social network support | -0.77(-1.74, 0.22) | NA |
| Weekly church attendance * social network support | -0.73 (-1.91, 0.46) | NA |
|  |  |  |
| Yearly church attendance * aid from co-religionists | NA | 1.91 (-4.36, 8.23) |
| Monthly church attendance * aid from co-religionists | NA | 0.36 (-5.54, 6.31) |
| Weekly church attendance * aid from co-religionists | NA | 1.32 (-4.54, 7.24) |

| Table S9: Results of Bayesian regression models predicting focal child’s performance on Stage 1 Assessments (Models 1 and 2; n = 5,814). Models include interaction terms of frequency of mother’s ritual attendance and mother’s social network support (Model 1) and frequency of mother’s ritual attendance and mother’s aid from co-religionists (Model 2). | | |
| --- | --- | --- |
|  | **Model 1**  **Regression Estimate**  **(with 95% CI)** | **Model 2**  **Regression Estimate**  **(with 95% CI)** |
| Intercept | 10.58 (10.03, 11.12) | 10.59 (10.04, 11.15) |
| Focal child sbling number (centered) | -0.35 (-0.44, -0.25) | -0.34 (-0.44, -0.25) |
| Mother’s partner’s children in household (centered) | 0.12 (-0.22, 0.47) | 0.11 (-0.25, 0.46) |
| Mother's church attendance | |  |
| Yearly | 0.18 (-0.01, 0.37) | 0.17 (-0.02, 0.36) |
| Monthly | 0.16 (-0.15, 0.48) | 0.14 (-0.19, 0.47) |
| Weekly | 0.38 (-0.03, 0.78) | 0.29 (-0.21, 0.80) |
| Mother’s social network support (scaled) | 0.28 (0.14, 0.41) | 0.19 (0.10, 0.28) |
| Mother’s aid from co-religionists | 0.13 (-0.23, 0.50) | -1.05 (-2.54, 0.39) |
| Mother’s weekly hours of work (centered) | -0.14 (-0.21, -0.08) | -0.14 (-0.20, -0.08) |
| Mother’s age (centered) | 0.04 (0.02, 0.06) | 0.04 (0.02, 0.06) |
| Mother’s education (centered) | 0.66 (0.57, 0.74) | 0.66 (0.58, 0.74) |
| Mother’s household income (centered) | 0.49 (0.41, 0.58) | 0.50 (0.41, 0.58) |
| Focal child sex (1=male, 0=female) | -0.99 (-1.15, -0.82) | -0.99 (-1.15, -0.82) |
| Mother’s partner present | -0.20 (-0.50, 0.11) | -0.20 (-0.52, 0.11) |
| Focal child ethnicity (1=white, 0=non-white) | -0.08 (-0.54, 0.38) | -0.09 (-0.57, 0.39) |
|  |  |  |
| Yearly church attendance * social network support | -0.12 (-0.32, 0.07) | NA |
| Monthly church attendance * social network support | -0.12 (-0.42, 0.18) | NA |
| Weekly church attendance * social network support | -0.29 (-0.59, 0.02) | NA |
|  |  |  |
| Yearly church attendance * aid from co-religionists | NA | -1.22 (-0.47, 2.92) |
| Monthly church attendance * aid from co-religionists | NA | -1.22 (-0.32, 2.83) |
| Weekly church attendance * aid from co-religionists | NA | -1.26 (-0.31, 2.82) |

| Table S10: Results of Bayesian regression models predicting focal child’s performance on WISQ-III Scores (Models 1 and 2; n = 5,379). Models include interaction terms of frequency of mother’s ritual attendance and mother’s social network support (Model 1) and frequency of mother’s ritual attendance and mother’s aid from co-religionists (Model 2). | | |
| --- | --- | --- |
|  | **Model 1**  **Regression Estimate**  **(with 95% CI)** | **Model 2**  **Regression Estimate**  **(with 95% CI)** |
| Intercept | 105.23 (102.47, 107.98) | 105.32 (102.65, 107.99) |
| Focal child sbling number (centered) | -1.36 (-1.83, -0.90) | -1.35 (-1.83, -0.87) |
| Mother’s partner’s children in household (centered) | 0.30 (-1.48, 2.10) | 0.31 (-1.51, 2.07) |
| Mother's church attendance | |  |
| Yearly | -0.16 (-1.12, 0.79) | -0.21 (-1.16, 0.77) |
| Monthly | 0.29 (-1.22, 1.77) | 0.08 (-1.47, 1.62) |
| Weekly | -0.63 (-2.49, 1.23) | -0.86 (-3.23, 1.46) |
| Mother’s social network support (scaled) | 0.95 (0.24, 1.68) | 0.61 (0.18, 1.05) |
| Mother’s aid from co-religionists | 2.45 (0.76, 4.08) | -3.68 (-11.38, 4.38) |
| Mother’s weekly hours of work (centered) | -0.68 (-0.99, -0.38) | -0.68 (-0.98, -0.37) |
| Mother’s age (centered) | 0.28 (0.18, 0.38) | 0.28 (0.18, 0.38) |
| Mother’s education (centered) | 3.95 (3.55, 4.35) | 3.95 (3.56, 4.36) |
| Mother’s household income (centered) | 1.95 (1.51, 2.39) | 1.95 (1.50, 2.39) |
| Focal child sex (1=male, 0=female) | 0.17 (-0.65, 0.99) | 0.16 (-0.66, 0.98) |
| Mother’s partner present | -2.10 (-3.81, -0.46) | -2.10 (-3.79, -0.44) |
| Focal child ethnicity (1=white, 0=non-white) | 0.02 (-2.27, 2.31) | -0.02 (-2.19, 2.19) |
|  |  |  |
| Yearly church attendance * social network support | -0.45 (-1.41, 0.50) | NA |
| Monthly church attendance * social network support | -0.16 (-1.57, 1.24) | NA |
| Weekly church attendance * social network support | -1.04 (-2.52, 0.38) | NA |
|  |  |  |
| Yearly church attendance * aid from co-religionists | NA | 5.37 (-3.63, 14.14) |
| Monthly church attendance * aid from co-religionists | NA | 6.86 (-1.68, 15.01) |
| Weekly church attendance * aid from co-religionists | NA | 6.14 (-2.35, 14.38) |

|  | Model  Regression Estimate  (with 95% CI) |
| --- | --- |
| Intercept | 0.20(-0.16, 0.55) |
| Year | 0.07 (0.01, 0.13) |
| Mother’s partner’s children in household (centered) | 0.18 (0.12, 0.24) |
| Mother's church attendance | |
| Yearly | -0.01 (-0.05, 0.03) |
| Monthly | 0.02 (-0.04, 0.08) |
| Weekly | 0.04 (-0.03, 0.11) |
| Mother’s religious affiliation | -0.02 (-0.07, 0.03) |
| Mother’s religious belief in God (ref: none) |  |
| Not sure | 0.03 (-0.02, 0.08) |
| Yes | 0.02 (-0.03, 0.08) |
| Mother’s social network support (scaled) | -0.02 (-0.04, -0.01) |
| Mother’s aid from co-religionists | 0.08 (0.02, 0.14) |
| Mother’s weekly hours of work (centered) | 0.00 (-0.01, 0.00) |
| Mother’s age (centered) | 0.02 (0.01, 0.02) |
| Mother’s education (centered) | -0.04 (-0.06, -0.03) |
| Mother’s household income (centered) | 0.00 (-0.02, 0.01) |
| Focal child sex (1=male, 0=female) | 0.02 (-0.01, 0.05) |
| Mother’s partner present | 0.05 (-0.01, 0.12) |
| Focal child ethnicity (1=white, 0=non-white) | 0.03 (-0.05, 0.11) |

**Table S11: Results of a Baysian regression model predicting a woman’s total number of children over 10 years. This model was a preliminary model run to examine the relative contribution of different religion variables to a mother’s fertility (n = 5,613).**

| Table S12: Results of Bayesian regression models predicting total offspring. Model 1 (n = 8,207) represents the results of our standard imputation strategy,  and is the same as is reported in Model 1 of Supplementary Table 5. Model 2 (n = 10.014) indicates the results of our key predictor variables, without any  imputation (as described in the Supplementary Methods Section 1.1.1). Model 3 (n = 13,433) indicates the results of the same model structure as Model 1,  but using an alternative imputation strategy implemented in Amelia (as described in Supplementary Methods Section 1.1.2.) | | | |
| --- | --- | --- | --- |
|  | **Model 1**  **Regression Estimate**  **(with 95% CI)** | **Model 2**  **Regression Estimate**  **(with 95% CI)** | **Model 3**  **Regression Estimate**  **(with 95% CI)** |
| Intercept | 0.16 (-0.18, 0.51) | -0.29 (-0.32, -0.26) | 0.36 (0.12, 0.60) |
| Year | 0.07 (0.01, 0.13) | NA | 0.09 (0.05, 0.13) |
| Mother’s partner’s children in household (centered) | 0.07 (0.04, 0.09) | NA | 0.06 (0.05, 0.07) |
| Mother's church attendance | |  |  |
| Yearly | 0.01 (-0.01, 0.02) | 0.03 (-0.02, 0.08) | 0.03 (0.02, 0.04) |
| Monthly | 0.04 (0.01, 0.06) | 0.11 (0.02, 0.21) | 0.05 (0.04, 0.06) |
| Weekly | 0.07 (0.04, 0.10) | 0.19 (0.07, 0.29) | 0.08 (0.07, 0.09) |
| Mother’s social network support (scaled) | -0.02 (-0.03, -0.01) | -0.15 (-0.17, -0.13) | -0.03 (-0.03, -0.03) |
| Mother’s aid from co-religionists | 0.04 (0.02, 0.07) | 0.18 (0.08, 0.27) | 0.03 (0.02, 0.04) |
| Mother’s weekly hours of work (centered) | 0.00 (0.00, 0.00) | NA | 0.00 (0.00, 0.00) |
| Mother’s age (centered) | 0.02 (0.02, 0.02) | NA | 0.02 (0.02, 0.02) |
| Mother’s education (centered) | -0.04 (-0.05, -0.03) | NA | -0.05 (-0.05, -0.05) |
| Mother’s household income (centered) | 0.00 (-0.01, 0.01) | NA | 0.00 (-0.01, 0.00) |
| Focal child sex (1=male, 0=female) | 0.01 (-0.01, 0.02) | NA | 0.01 (0.00, 0.01) |
| Mother’s partner present | 0.07 (0.04, 0.09) | NA | 0.00 (-0.01, 0.02) |
| Focal child ethnicity (1=white, 0=non-white) | 0.05 (0.00, 0.09) | NA | -0.02 (-0.03, 0.00) |

**3. Supplementary References**

Honaker, J., King, G., & Blackwell, M. (2011). Amelia II: A program for missing data. *Journal of statistical software*, *45*(7), 1-47.
